# SMAD4 induces opposite effects on metastatic growth from pancreatic tumors depending on the organ of residence

**DOI:** 10.1038/s43018-025-01047-5

**Published:** 2025-09-25

**Authors:** Kaloyan M. Tsanov, Francisco M. Barriga, Yu-Jui Ho, Direna Alonso-Curbelo, Geulah Livshits, Sha Tian, Richard P. Koche, Timour Baslan, Janelle Simon, Alexandra N. Wuest, José Reyes, Jin Park, Wei Luan, John E. Wilkinson, Umesh Bhanot, Jordana Ray-Kirton, Ignas Masilionis, Nevenka Dimitrova, Christine A. Iacobuzio-Donahue, Ronan Chaligné, Dana Pe’er, Joan Massagué, Scott W. Lowe

**Affiliations:** 1 Cancer Biology & Genetics Program, Sloan Kettering Institute, Memorial Sloan Kettering Cancer Center, New York, NY USA; 2 Section of Hematology/Oncology, Department of Medicine, The University of Chicago, Chicago, IL USA; 3 Ben May Department for Cancer Research, The University of Chicago, Chicago, IL USA; 4 Systems Oncology Program, Vall d’Hebron Institute of Oncology (VHIO), Vall d’Hebron Barcelona Hospital Campus, Barcelona, Spain; 5 Institute for Research in Biomedicine (IRB), The Barcelona Institute of Science and Technology (BIST), Barcelona, Spain; 6 Center for Epigenetics Research, Memorial Sloan Kettering Cancer Center, New York, NY USA; 7 Department of Biomedical Sciences, School of Veterinary Medicine, The University of Pennsylvania, Philadelphia, PA USA; 8 Computational & Systems Biology Program, Sloan Kettering Institute, Memorial Sloan Kettering Cancer Center, New York, NY USA; 9 Department of Pathology, University of Michigan School of Medicine, Ann Arbor, MI USA; 10 Pathology Core Facility, Department of Pathology and Laboratory Medicine, Memorial Sloan Kettering Cancer Center, New York, NY USA; 11 David M. Rubenstein Center for Pancreatic Cancer Research, Department of Pathology and Laboratory Medicine, Memorial Sloan Kettering Cancer Center, New York, NY USA; 12 Howard Hughes Medical Institute, Memorial Sloan Kettering Cancer Center, New York, NY USA

**Keywords:** Metastasis, Cancer epigenetics, Pancreatic cancer, Cancer

## Abstract

The role of driver gene mutations in sustaining tumor growth at metastatic sites is poorly understood. *SMAD4* inactivation is a paradigm of such mutations and a hallmark of pancreatic ductal adenocarcinoma (PDAC). To determine whether metastatic tumors are dependent on *SMAD4* inactivation, we developed a mouse model of PDAC that enables spatiotemporal control of *Smad4* expression. While *Smad4* inactivation in the premalignant pancreas facilitated the formation of primary tumors, *Smad4* reactivation in metastatic disease suppressed liver metastases but promoted lung metastases. These divergent effects were underpinned by organ-biased differences in the tumor cells’ chromatin state that emerged in the premalignant pancreas and were distinguished by the dominance of KLF4 versus RUNX1 transcription factors. Our results show how epigenetic states favored by the organ of residence can influence the output of driver mutations in metastatic tumors, which has implications for interpreting tumor genetics and therapeutically targeting metastatic disease.

## Abstract (web summary)

Using mouse pancreatic cancer models, Tsanov et al. show that reactivation of *SMAD4* at metastatic sites promotes tumor growth in the lung through RUNX1 but restrains metastatic growth through KLF4 in the liver, influenced by organ-specific epigenetic states.

## Main

Metastatic disease—the growth of cancers beyond the primary tumor—accounts for 90% of cancer-related deaths^1^. Metastasis involves the acquisition of multiple traits that enable cells to leave the primary tumor, survive in the circulation and ultimately reach and colonize other organs^1,2^. Despite the distinct capabilities that must be acquired for a tumor cell to successfully metastasize, genome-sequencing efforts have identified few driver gene mutations that are specific to metastatic tumors^3–5^. This suggests that prometastatic traits arise from epigenetic programs that facilitate cell state changes such as epithelial-to-mesenchymal transitions (EMTs)^1,2,6^. Although driver gene mutations can endow primary tumors with increased metastatic capacity^7,8^, whether or how tumor evolution or the metastatic site itself influences their contribution to tumor maintenance is unknown. Such knowledge would have important implications for precision oncology and may guide the development of much needed metastasis-targeting therapies.

Among driver mutations, inactivation of the *SMAD4* tumor suppressor gene—a core mediator of transforming growth factor-β (TGFβ) signaling—is a hallmark of several gastrointestinal malignancies that is found at the highest frequency in pancreatic ductal adenocarcinoma (PDAC)^5,9,10^. During cancer progression, *SMAD4* inactivation shifts TGFβ’s activity from tumor-suppressive to tumor-promoting by impairing its ability to trigger cell-cycle arrest and EMT-coupled apoptosis^11,12^. Accordingly, *SMAD4*-mutant tumors have higher rates of metastasis in PDAC patients, an effect recapitulated in animal models^13–16^. Despite its clear role in facilitating progression of primary tumors, it is unknown whether *SMAD4* inactivation is required to sustain disease at metastatic sites. This represents an important knowledge gap when considering strategies to target the TGFβ pathway in PDAC patients, who almost invariably present with metastatic disease^17^. In this study, we took advantage of a murine model that enables inducible and reversible *Smad4* inactivation at different PDAC stages to interrogate the ongoing need for *Smad4* inactivation in metastatic disease. Our results reveal a diametrically opposed role for *Smad4* inactivation in sustaining liver and lung metastases and establish a critical interplay between driver mutations and organ-biased chromatin states that contributes to the heterogeneity of cancers driven by identical genetic lesions.

## Results

### *Smad4*-restorable genetically engineered mouse model (GEMM) of PDAC

**Fig. 1:**
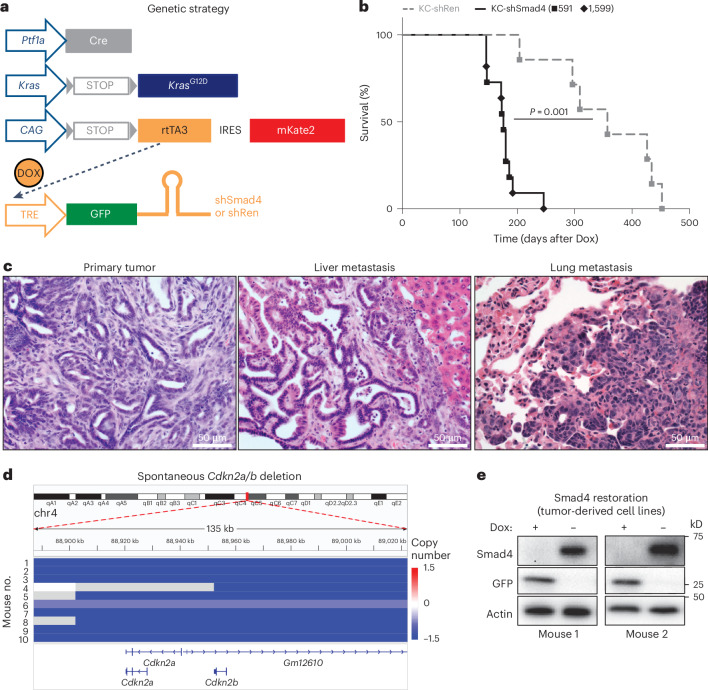
A *Smad4*-restorable GEMM of PDAC. **a**, Schematic of GEMM alleles. rtTA3, third-generation reverse tetracycline transactivator; TRE, tetracycline response element. **b**, Overall survival of KC-shRen mice and KC-shSmad4 mice (expressing one of two independent Smad4 shRNAs: 591 or 1599) after Dox administration (*n* = 7 KC-shRen mice; *n* = 11 KC-shSmad4 mice). Statistical analysis was conducted using a log-rank test. **c**, H&E staining of primary tumors and liver and lung metastases from KC-shSmad4 mice. Data are representative of ten, six and three independent mice, respectively. **d**, sWGS analysis of the *Cdkn2a/b* locus in KC-shSmad4 tumor-derived cell lines (*n* = 10 independent mice). **e**, Western blot analysis of Dox response in vitro. Data are representative of six independent KC-shSmad4 cell lines. Source Data

To enable reversible *Smad4* inactivation in PDAC, we generated a GEMM that harbors pancreas-specific, single-copy, doxycycline (Dox)-inducible short hairpin RNA (shRNA) against *Smad4* (or against *Renilla* luciferase as a control) on the background of oncogenic *Kras*^G12D^ (hereafter KC-shSmad4 and KC-shRen, respectively; Methods) (Fig. 1a). This genetic strategy allows for tumor development in the setting of *Smad4* depletion (through Dox administration) and subsequent restoration of *Smad4* expression at physiological levels from its endogenous locus (through Dox withdrawal). The alleles also contain two fluorescent reporters that facilitate identification and isolation of tumor cells: a constitutive mKate2 and an shRNA-linked GFP (Fig. 1a).

**Extended Data Fig. 1:**
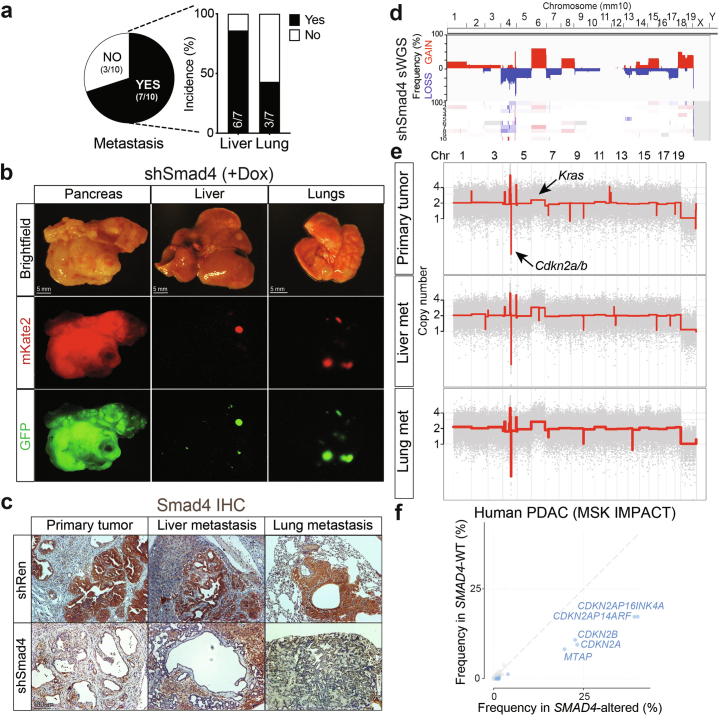
Characterization of the KC-shSmad4 mouse model. (**a**) Overall (pie chart) and organ-specific (stacked bar graph) frequency of metastasis in KC-shSmad4 mice. (**b**) Macroscopic images of tumor-bearing pancreas, liver, and lungs from KC-shSmad4 mice ( + Dox) at endpoint. Fluorescent images match the corresponding brightfield images. Data are representative of 10 (pancreas), 6 (liver), and 3 (lungs) independent mice. (**c**) Immunohistochemistry (IHC) staining for SMAD4 in primary and metastatic KC-shSmad4 tumors ( + Dox). Dashed lines demarcate metastases. Data are representative of 3 independent mice. (**d**) sWGS analysis of genome-wide copy number alterations in KC-shSmad4 primary tumor-derived cell lines (n = 10 independent mice). Frequency plot is shown on the top and individual sample tracks are provided on the bottom. (**e**) sWGS analysis of genome-wide copy number alterations in KC-shSmad4 tumor-derived cell lines from matched primary tumors, liver and lung metastases. The *Kras* and *Cdkn2a/b* loci are highlighted (arrows). Data are representative of 3 independent mice. (**f**) Frequency of the indicated homozygous deletions in *SMAD4*-altered (mutated or homozygously deleted, n = 959) or wild-type (WT, n = 3188) PDAC tumors in the MSK-IMPACT cohort (*CDKN2A/B*, p < 10^−10^; q < 10^−10^). *CDKN2A/B* and their adjacent gene *MTAP* are highlighted. Source Data

Consistent with results from conventional knockout GEMMs^16,18–20^, *Smad4* depletion (by continuous Dox administration starting at 5 weeks of age) promoted tumor initiation, shortened survival and led to the development of tumors that metastasize to the liver and, less frequently, to the lungs (Fig. 1b,c and Extended Data Fig. 1a). At the late stage, primary and metastatic tumors expressed the fluorescent reporters (Extended Data Fig. 1b) and maintained potent depletion of SMAD4 protein (Extended Data Fig. 1c). Notably, tumor formation appeared to require further inactivation of the *Cdkn2a* tumor suppressor gene, as sparse whole-genome sequencing (sWGS) of tumor-derived cell lines revealed spontaneous homozygous deletion of the *Cdkn2a/b* locus in nine of ten cases^21^ (Fig. 1d). This lesion (along with *Kras* gain) was the most prominent event in an otherwise largely stable genome (Extended Data Fig. 1d), in agreement with the previously reported genome evolution of PDAC driven by alterations in the TGFβ pathway^22^. The *Cdkn2a/b* deletions were highly concordant between primary and metastatic tumors (Extended Data Fig. 1e) and they mirrored the genetic association of *SMAD4* alterations with homozygous *CDKN2A/B* deletions in the MSK-IMPACT cohort of human PDAC (Extended Data Fig. 1f). Thus, the KC-shSmad4 GEMM recapitulates cardinal features of the human disease and further enables reversible *Smad4* inactivation.

### Organ-specific effects of *Smad4* restoration on tumor growth

**Extended Data Fig. 2:**
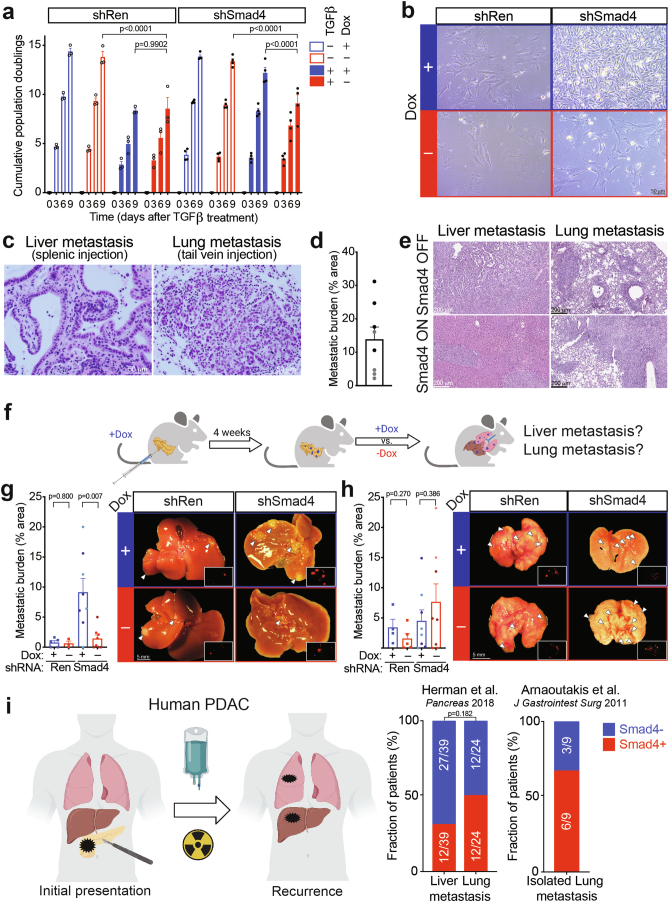
*Smad4* restoration *in vitro* and in murine liver and lung metastases, and SMAD4 expression in human metastatic recurrence. (**a**) Proliferation assays of KC-shRen and KC-shSmad4 cells +/-Dox +/-TGFβ *in vitro* (mean + SEM; n = 3 and 4 independent cell lines per genotype, respectively). (**b**) Brightfield images of KC-shRen and KC-shSmad4 cells after TGFβ treatment +/-Dox for 6 days. Data represent 3 independent cell lines per genotype. (**c**) H&E staining of liver and lung metastases generated by intrasplenic or tail vein injections of KC-shSmad4 cell lines. Data represent 4 independent mice per organ. (**d**) Percent-area quantification of liver metastasis burden at day 0 of Dox withdrawal after intrasplenic injections of KC-shSmad4 cell (n = 8 independent mice). Different color shading indicates independent cell lines. (**e**) H&E staining of liver and lung metastases from KC-shSmad4 cells without (Smad4 OFF) or with *Smad4* restoration (Smad4 ON). Data are representative of 6 independent mice per organ. (**f**) Schematic of orthotopic experiments with KC-shRen and KC-shSmad4 cells for analysis of metastasis burden. Created in BioRender. Tsanov, K. (2025) https://BioRender.com/yihgg0q. (**g**) Analysis of liver metastasis burden after orthotopic injections of KC-shRen or KC-shSmad4 cells with or without subsequent Dox withdrawal. (Left) Percent-area quantifications at endpoint (mean + SEM; n = 5, 3, 8 and 8 independent mice for groups shown, left to right). (Right) Representative macroscopic images of tumor-bearing livers. (**h**) Analysis of lung metastasis burden after orthotopic injections of KC-shRen or KC-shSmad4 cells with or without subsequent Dox withdrawal. (Left) Percent-area quantifications at endpoint (mean + SEM; n = 4, 5, 8 and 8 independent mice for groups shown, left to right). (Right) Representative macroscopic images of tumor-bearing lungs. Different color shading in (**g-h**) indicates independent cell lines, arrowheads highlight metastatic tumors, and insets show mKate2 reporter. (**i**) (Left) Schematic of metastasis recurrence studies in human PDAC^25,26^. Created in BioRender. Tsanov, K. (2025) https://BioRender.com/dt9i4jd. (Right) Fraction of patients whose tumors stained SMAD4-positive or -negative by IHC, according to site of recurrence. Exact numbers are indicated on each bar. Statistical analyses were two-way ANOVA (**a**), unpaired two-sided t-test (**g, h**), and Fisher’s exact test (**i**). Source Data

To study *Smad4* reactivation in metastatic tumors, we turned to transplantation assays using cell lines derived from primary GEMM tumors that were capable of metastasis, as this approach afforded longer experimental time before tumor burden necessitated mouse euthanasia. The tumor-derived cell lines maintained robust *Smad4* restorability (Fig. 1e), mounted an expected *Smad4*-dependent cytostatic response to TGFβ in vitro (Extended Data Fig. 2a,b) and produced metastases with remarkably similar histopathology to those emerging in the GEMMs and in PDAC patients^23^ (Extended Data Fig. 2c). *Smad4*-dependent apoptotic responses induced by TGFβ in PDAC epithelial progenitors^11,24^ were not captured in these cell lines at the analyzed time points.

**Fig. 2:**
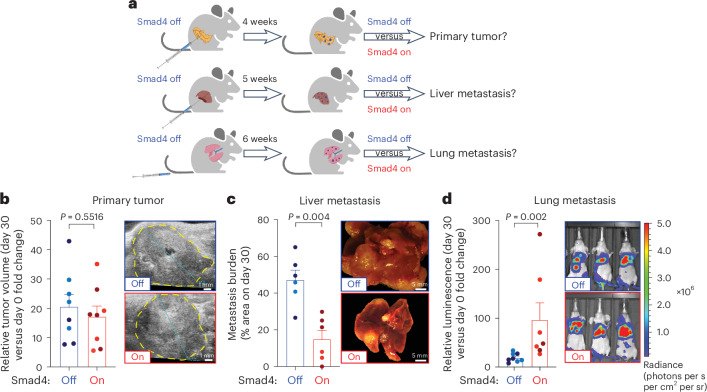
*Smad4* restoration has organ-specific effects on tumor growth. **a**, Schematic of orthotopic, intrasplenic and tail-vein injection experiments. This image was created using BioRender.com. **b**, Analysis of primary tumor growth after orthotopic transplantation of KC-shSmad4 cells and subsequent *Smad4* restoration. Left, fold-change quantifications of tumor volume on day 30 versus day 0 of Dox withdrawal (mean ± s.e.m.; *n* = 8 independent mice per group). Different color shading indicates independent cell lines. Right, representative ultrasound images of tumors (demarcated by dashed yellow lines). **c**, Analysis of liver metastasis burden after intrasplenic injections of KC-shSmad4 cells and subsequent *Smad4* restoration. Left, percentage area quantifications on day 30 after Dox withdrawal (mean ± s.e.m.; *n* = 6 independent mice per group). Different color shading indicates independent cell lines. Right, representative macroscopic images of tumor-bearing livers. **d**, Analysis of lung metastasis burden after tail-vein injections of KC-shSmad4 cells and subsequent *Smad4* restoration. Left, fold-change quantifications of bioluminescence signal on day 30 versus day 0 of Dox withdrawal (mean ± s.e.m.; *n* = 8 and 7 independent mice per group, respectively). Different color shading indicates independent cell lines. Right, representative bioluminescence images of tumor-bearing mice. Statistical analyses were conducted using an unpaired two-sided *t*-test (**b**) or two-sided Mann–Whitney *U*-test (**c**,**d**). Source Data

KC-shSmad4 cells were stably transduced with firefly luciferase to facilitate tumor monitoring and delivered through orthotopic, intrasplenic or tail-vein injections into nude mice to respectively generate primary tumors, liver metastases or lung metastases (Fig. 2a). After 4–6 weeks, which allowed for tumor formation under *Smad4*-depleted conditions (+Dox = Smad4 off), *Smad4* expression was restored by Dox withdrawal (−Dox = Smad4 on) in a randomly selected half of each cohort and tumor burden was assessed 30 days later (Fig. 2a). Strikingly, the response to *Smad4* restoration was different in each of the three organs; tumor burden was unchanged in the pancreas, decreased in the liver and increased in the lungs (Fig. 2b–d and Extended Data Fig. 2d,e). Of note, similar results were obtained in spontaneous metastases arising from the orthotopic KC-shSmad4 transplants but not in KC-shRen cells, thus ruling out artifactual effects because of the used metastasis assays or Dox administration (Extended Data Fig. 2f–h).

To determine the relevance of our findings to human PDAC, we queried data on metastatic recurrence in PDAC patients after primary tumor removal, where *SMAD4* status was evaluated by immunohistochemistry (IHC) staining of resected primary or metastatic tumors^25,26^. Corroborating a potentially tumor-suppressive versus tumor-promoting function of *SMAD4* in the liver versus lungs, 69% of cases with recurrent liver metastases lacked SMAD4 expression, in contrast to 50% of concurrent and 33% of isolated lung metastases (Extended Data Fig. 2i). Thus, *Smad4* inactivation confers a selective advantage to liver metastases and a disadvantage to lung metastases, consistent with SMAD4 protein expression patterns in PDAC patients with metastatic recurrence.

### Distinct *Smad4* transcriptional outputs in liver versus lung

**Extended Data Fig. 3:**
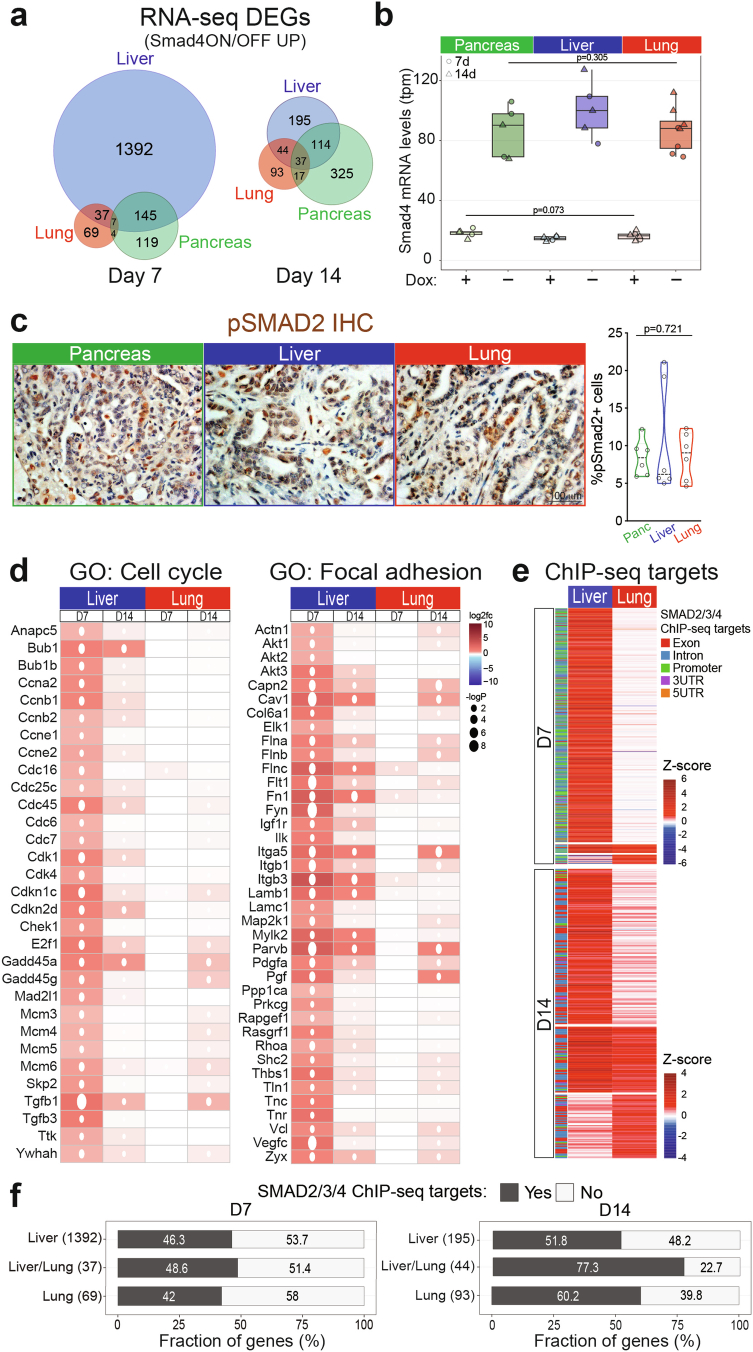
Effects of *Smad4* restoration on gene expression and signaling. (**a**) Overlap between upregulated genes in tumor cells from the pancreas, liver, or lungs at 7 and 14 days after Dox withdrawal. Numbers reflect genes in each category. Complete gene lists are provided in Supplementary Table 1. DEGs = differentially expressed genes. (**b**) Normalized *Smad4* mRNA levels (RNA-seq data; tpm = transcripts per million) in KC-shSmad4 tumor cells from the pancreas, liver, or lungs +/-Dox for 7 or 14 days (n = 5, 5, 6, 5, 6 and 8 independent mice for groups shown, left to right). Box plots represent median +/- upper/lower quartile (box) +/-1.5 x IQR between the first and third quartile. (**c**) Representative IHC staining for pSMAD2 in KC-shSmad4 tumors ( + Dox) from the pancreas, liver, or lungs. Quantifications (%pSMAD2 cells) are shown on the right (n = 6 independent primary tumor regions or metastatic tumors from 2 mice per organ). (**d**) Heatmap of differentially expressed genes from the indicated KEGG pathways. Average log2 fold-change and p-values are shown for each organ and timepoint. (**e**) Heatmap of SMAD4-dependent SMAD2/3 ChIP target genes that are differentially upregulated in liver vs. lung metastases at 7 or 14 days after Dox withdrawal. Average RNA-seq log_2_ fold-change values are shown for Smad4 ON vs. OFF in each organ and timepoint. The type of genomic binding region is color-annotated on the left. Complete gene lists are provided in Supplementary Table 1. (**f**) Fraction of upregulated genes in liver and/or lung metastases at 7 or 14 days of Dox withdrawal that are SMAD4-dependent SMAD2/3 ChIP-seq targets. Absolute number of genes per group is shown in parentheses. Statistical analyses were one-way ANOVA (**b, c**), and Wald test with negative binomial modeling and Benjamini-Hochberg adjustment (**d**). Source Data

SMAD4 acts as a transcription factor (TF) by forming a complex with the SMAD2/3 TFs to activate gene expression programs downstream of TGFβ receptors^27^. Hence, we performed RNA sequencing (RNA-seq) to explore the transcriptional basis of the observed organ-specific phenotypes. Leveraging the mKate2 reporter in our model, we used fluorescence-activated cell sorting (FACS) to isolate tumor (mKate2^+^) cells 7 and 14 days after Dox withdrawal, which allowed for assessment of *Smad4* output kinetics. Consistent with its organ-specific effects on tumor growth, *Smad4* restoration led to upregulation of partially overlapping but mostly distinct genes, as compared to tumor cells kept on Dox: 89% (1,580/1,773) and 74% (613/825) organ-specific genes on days 7 and 14, respectively (Extended Data Fig. 3a and Supplementary Table 1). This phenomenon was particularly pronounced in the liver at the 7-day time point and was still observed across all three organs on day 14 (Extended Data Fig. 3a). Importantly, these results were not confounded by differential *Smad4* expression or baseline TGFβ signaling, as the extent of *Smad4* depletion and restoration and the levels of phosphorylated SMAD2 (a SMAD4-independent readout of TGFβ signaling^27^) were similar among the three organs (Extended Data Fig. 3b,c).

**Fig. 3:**
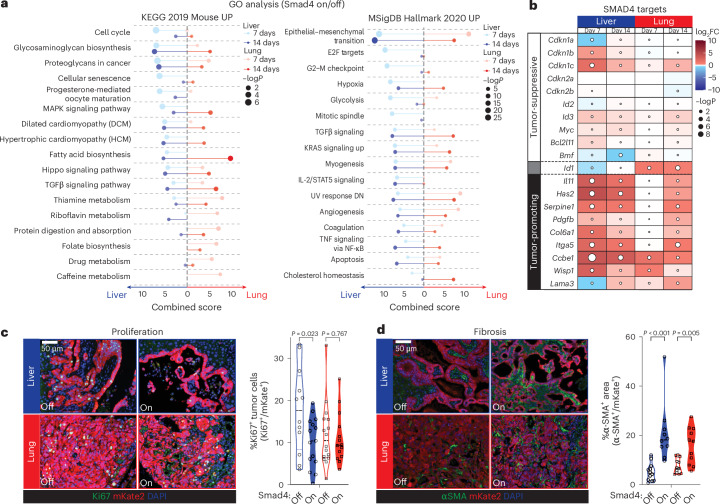
*Smad4* induces different transcriptional programs in liver versus lung metastases. **a**, GO analysis of upregulated genes in liver or lung metastases 7 or 14 days after Dox withdrawal. Combined scores and *P* values for the top KEGG (left) or Hallmark (right) pathways are shown for Smad4 on versus off for each organ and time point. Complete GO lists are provided in Supplementary Table 2. **b**, Heat map of representative genes from *Smad4*’s cytostatic or apoptotic (tumor-suppressive) and fibrogenic (tumor-promoting) transcriptional programs. The average RNA-seq log_2_ fold change (FC) and *P* values are shown for Smad4 on versus off for each organ and time point. **c**, Representative IF staining for Ki67 in KC-shSmad4 liver and lung metastases ± *Smad4* restoration. mKate2 was used to label tumor cells. Right, quantifications (*n* = 12, 15, 16 and 14 independent tumors from three, four, three and three mice for the groups shown, from left to right). Analysis was performed 7 days after Dox withdrawal. **d**, Representative IF staining for α-SMA in KC-shSmad4 liver and lung metastases ± *Smad4* restoration. mKate2 was used to label tumor cells. Right, quantifications (*n* = 12, 10, 12 and 11 independent tumors from three, three, three and four mice for the groups shown, from left to right). Analysis was performed at the experimental endpoint (30 days for liver; 45 days for lungs). Statistical analyses were conducted using Fisher’s exact test with Benjamini–Hochberg adjustment (**a**), a Wald test with negative binomial modeling and Benjamini–Hochberg adjustment (**b**), an unpaired two-sided *t*-test (**c**) or a two-sided Mann–Whitney *U*-test (**d**). Source Data

To better understand the organ-specific consequences of *Smad4* restoration, we next performed functional annotation of *Smad4*-activated genes in the liver and lungs, as these organs exhibited opposite tumor growth phenotypes. Gene ontology (GO) analysis revealed that tumors in both organs upregulated extracellular matrix (for example, glycosaminoglycan, proteoglycan and focal adhesion) and EMT-related transcriptional programs, while only liver metastases showed enrichment for gene signatures related to the cell cycle and senescence (Fig. 3a, Extended Data Fig. 3d and Supplementary Table 2). Intersection of these gene lists with available data from SMAD2/3 ChIP-seq (chromatin immunoprecipitation followed by sequencing) of murine PDAC cells^11,24^ confirmed differential engagement of SMAD4-dependent binding targets, further implicating direct effects of *Smad4* reactivation (Extended Data Fig. 3e,f). Of note, GO analysis of RNA-seq data from primary tumors showed enrichment of only a few pathways, which did not include cell cycle or senescence (Supplementary Table 2), in line with the unchanged tumor burden in the pancreas.

**Extended Data Fig. 4:**
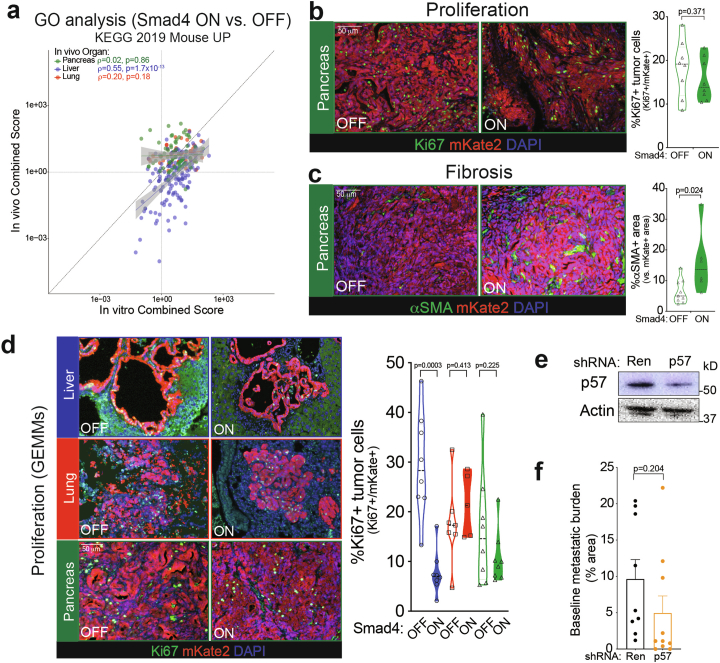
Impact of *Smad4* restoration on pathways *in vitro* vs. *in vivo*, on primary tumor phenotypes, and in GEMMs. (**a**) Comparison of gene ontology (GO) analyses of upregulated genes after Dox withdrawal *in vitro* (x axis) and *in vivo* in the pancreas, liver or lungs (y axis). See Methods for experimental details. Combined scores, correlation coefficients (ρ) and correlation p-values for the enriched KEGG pathways are shown for each organ. Complete GO lists and combined scores are provided in Supplementary Table 2. (**b**) Representative immunofluorescence staining for Ki67 in KC-shSmad4 primary tumors +/-*Smad4* restoration. mKate2 labels tumor cells. Quantifications are shown on the right (n = 8 independent tumor regions from two mice per group, respectively). Analysis was performed 7 days after Dox withdrawal. (**c**) Representative immunofluorescence staining for a-SMA in KC-shSmad4 primary tumors +/-*Smad4* restoration. mKate2 labels tumor cells. Quantifications are shown on the right (n = 9 and 6 independent tumor regions from two mice per group, respectively). Analysis was performed at experimental endpoint (45 days after Dox withdrawal). (**d**) Representative immunofluorescence staining for Ki67 in KC-shSmad4 GEMM primary tumors and liver and lung metastases +/-*Smad4* restoration. mKate2 labels tumor cells. Quantifications are shown on the right (n = 8, 8, 7, 5, 8 and 8 independent primary tumor regions or metastatic tumors from 3, 4, 4, 3, 3 and 3 mice for groups shown, left to right). Analysis was performed 7 days after Dox withdrawal. (**e**) Western blot analysis of shRNA efficiency. *Smad4*-restored cells were pre-treated with TGFb to induce p57 expression. Actin was used as a loading control. (**f**) Quantifications of baseline metastasis burden (% tumor area) after intrasplenic injections of KC-shSmad4 cells under continuous Dox administration (mean + SEM; n = 9 and 10 independent mice per group, respectively). Livers were harvested 68 days after injection. Statistical analyses were Pearson correlation (**a**), and unpaired two-sided t-test (**b, c, d, f**). Source Data

We also performed RNA-seq of the input cells in vitro after *Smad4* restoration and TGFβ treatment (Methods) and compared the resulting pathway enrichment to the in vivo data from the pancreas, liver and lungs (Extended Data Fig. 4a). There was partial overlap between the pathways enriched in vitro and in vivo, which showed the strongest correlation for the liver (Extended Data Fig. 4a), and key pathways (for example, focal adhesion, proteoglycans and cellular senescence) appeared to be better engaged in vivo according to the fraction of upregulated genes per pathway (Supplementary Table 2). Thus, multiple pathways are accessible and responsive to TGFβ in vitro but they become fully deployed at the respective metastatic site in vivo.

Importantly, the differentially expressed SMAD4 targets in the liver and lungs included genes that distinguish between SMAD4’s tumor-suppressive and tumor-promoting functions. They prominently featured upregulation of the tumor suppressor gene and cell-cycle inhibitor *Cdkn1c* (also known as p57^KIP2^)^28,29^ specifically in the liver, as well as induction of SMAD4’s tumor-promoting fibrogenic program (including *Il11*, *Has2*, *Serpine1*, *Col6a1*, *Itga5*, *Ccbe1* and *Wisp1*)^24^ in both organs, albeit with a delayed kinetics in the lungs (Fig. 3b). Of note, the SMAD4-dependent target *Id1*, known to reflect TGFβ’s protumor mode of action^30^, showed elevated expression in lung metastases but downregulation in liver metastases (Fig. 3b).

These transcriptomic data suggesting differential cytostatic and fibrogenic outputs were validated by immunostaining for the proliferation marker Ki67 and the TGFβ-dependent myofibroblast marker α-smooth muscle actin (α-SMA)^24^. In support of a liver-specific cytostatic response and shared fibrogenic response, *Smad4* reactivation reduced the proportion of Ki67^+^ tumor cells only in the liver, while both the liver and lungs exhibited increases in α-SMA levels (Fig. 3c,d). Analogous analyses of primary tumors revealed an unchanged fraction of Ki67^+^ tumor cells (Extended Data Fig. 4b), consistent with the lack of a growth phenotype, and a milder increase in α-SMA levels (Extended Data Fig. 4c). Importantly, the liver-specific cytostatic effect of *Smad4* reactivation was confirmed in the GEMMs, where short-term (7-day) *Smad4* restoration reduced the percentage of Ki67^+^ tumor cells in liver metastases but not in primary tumors or lung metastases (Extended Data Fig. 4d). While we were unable to assess longer-term effects on fibrosis because of the limited time between reliable metastasis detection and humane endpoint, these data validate in an autochthonous model the discordance of a key *Smad4* output that underpins its organ-specific tumor phenotypes.

**Fig. 4:**
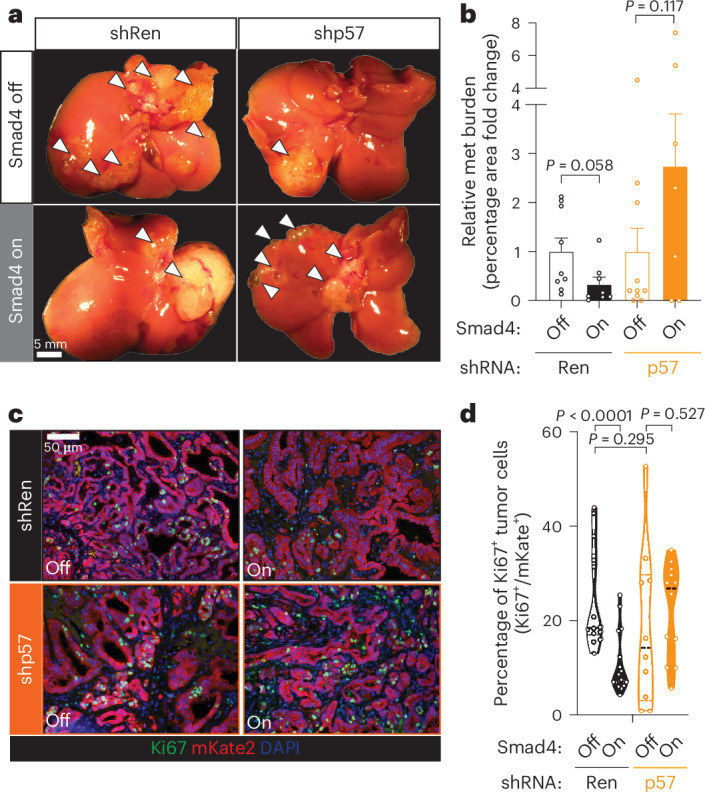
*Smad4*’s tumor-suppressive function depends on p57. **a**, Representative macroscopic images of tumor-bearing livers ± *Smad4* restoration on the background of the indicated stable shRNAs. Arrowheads highlight metastatic tumors. Data are representative of the number of independent mice per group specified in **b**. **b**, Quantifications of metastasis burden (fold change of percentage tumor area) on day 40 after Dox withdrawal (mean ± SEM; *n* = 9, 8, 10 and 7 independent mice per groups shown, from left to right). **c**,**d**, Representative IF staining (**c**) and quantifications (**d**) for Ki67 in KC-shSmad4 liver metastases ± *Smad4* restoration on the background of the indicated stable shRNAs (*n* = 14, 18, 10 and 12 independent tumors from three, four, three and four mice for groups shown, from left to right). Analysis was performed 7 days after Dox withdrawal. Statistical analysis was conducted using an unpaired two-sided *t*-test (**b**,**d**). Source Data

As noted above, one differentially expressed gene (DEG) that stood out was the cyclin-dependent kinase inhibitor p57 (*Cdkn1c*), a known target of SMAD4 that mediates TGFβ’s cytostatic function in certain contexts^29,31^, which showed preferential induction by *Smad4* in the liver (Fig. 3b). To probe p57 function in this context, we introduced shRNAs targeting p57 or a control gene (*Renilla* luciferase) in KC-shSmad4 cells, confirmed knockdown (Extended Data Fig. 4e) and performed intrasplenic injections to generate experimental liver metastases in mice on Dox (Smad4 off). At 30 days after injection, Dox was withdrawn to allow for *Smad4* re-expression and livers were assessed for macrometastases after another 40 days. Whereas tumors harboring the control shRNA showed the expected reduction in tumor burden (Fig. 4a,b) and fraction of Ki67^+^ cells (Fig. 4c,d) following *Smad4* reactivation, the p57-depleted tumors did not. Of note, these observations were specific to *Smad4* restoration and not a general effect of p57 knockdown on proliferation, as p57 depletion did not affect baseline tumor burden or the fraction of Ki67^+^ tumor cells in the *Smad4*-deficient condition (Fig. 4d and Extended Data Fig. 4f). Therefore, p57 is a crucial component of *Smad4*’s tumor-suppressive program in the liver. More broadly, our analysis reveals discordant engagement of *Smad4*’s tumor-suppressive versus tumor-promoting effectors in liver versus lung metastases, demonstrating that the anatomic site of the tumor can uncouple TGFβ’s antitumorigenic and protumorigenic programs.

### Distinct chromatin states in liver versus lung metastases

**Fig. 5:**
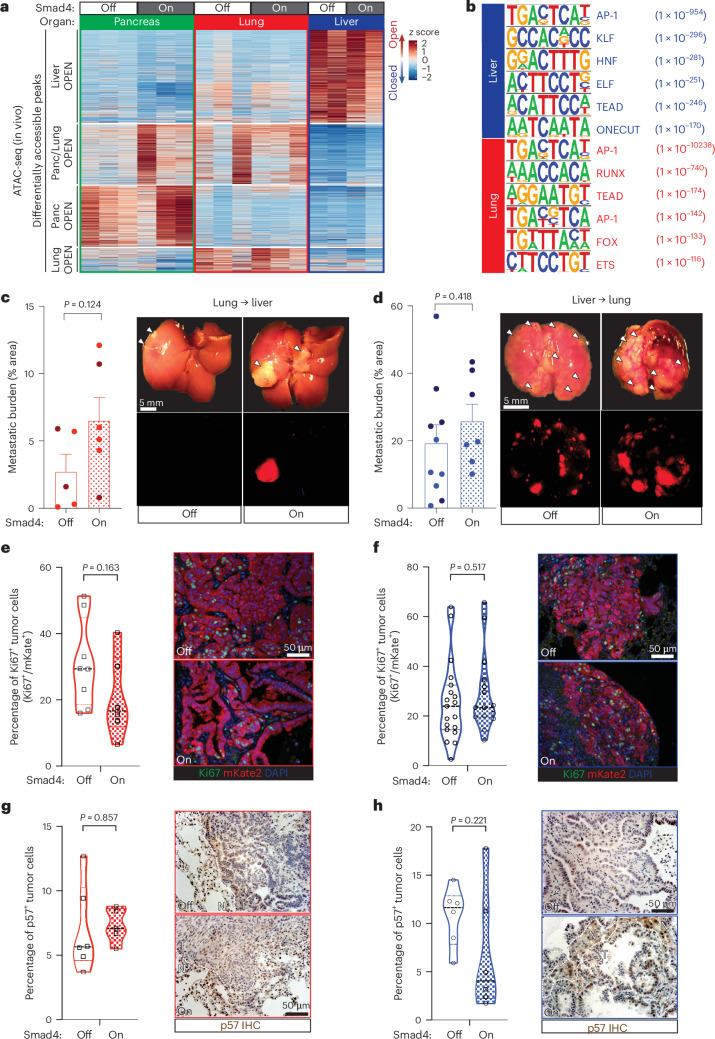
Liver and lung metastases harbor distinct chromatin states. **a**, Heat map of differentially accessible chromatin regions in tumor (mKate2^+^) cells isolated from the pancreas, liver or lungs (in vivo ATAC-seq). Smad4 on corresponds to 14 days of Dox withdrawal. Each column represents an independent mouse. A complete list of differentially accessible ATAC-seq peaks is provided in Supplementary Table 3. **b**, Top-scoring TF motifs identified by HOMER de novo motif analysis of in vivo ATAC-seq peaks enriched in liver versus lung metastases. Enrichment *P* values are shown in parentheses. **c**, Analysis of liver metastasis burden after intrasplenic injections of lung metastasis-derived KC-shSmad4 cell lines with or without subsequent Dox withdrawal. Left, percentage area quantifications at endpoint (mean ± s.e.m.; *n* = 5 and 6 independent mice per group, respectively; different color shading indicates independent cell lines). Right, representative macroscopic images of tumor-bearing livers. Shown are matching brightfield and mKate2 fluorescence images. Arrowheads highlight metastatic tumors. **d**, Analysis of lung metastasis burden after tail-vein injections of liver metastasis-derived KC-shSmad4 cell lines with or without subsequent Dox withdrawal. Left, percentage area quantifications at endpoint (mean ± s.e.m.; *n* = 10 and 7 independent mice per group, respectively; different color shading indicates independent cell lines). Right, representative macroscopic images of tumor-bearing lungs. Shown are matching brightfield and mKate2 fluorescence images. Arrowheads highlight metastatic tumors. **e**,**f**, Quantifications of the percentage of Ki67^+^ tumor cells in KC-shSmad4 lung-to-liver (**e**) and liver-to-lung (**f**) metastases ± *Smad4* restoration. Right, representative IF images (*n* = 8 and 6 independent tumors from two mice per respective group for liver; *n* = 19 and 15 independent tumors from five mice per respective group for lung). mKate2 was used to label tumor cells. Analysis was performed 7 days after Dox withdrawal. **g**,**h**, Quantifications of the percentage of p57^+^ cells in KC-shSmad4 lung-to-liver (**g**) and liver-to-lung (**h**) metastases ± *Smad4* restoration. Right, representative IHC images (*n* = 6 and 5 independent tumors from two mice per respective group for liver; *n* = 6 independent tumors from five mice per group for lung). mKate2 was used to label tumor cells. Analysis was performed 7 days after Dox withdrawal. Statistical analyses were conducted using a hypergeometric test (**b**) or unpaired two-sided *t*-test (**c**–**h**). Source Data

**Extended Data Fig. 5:**
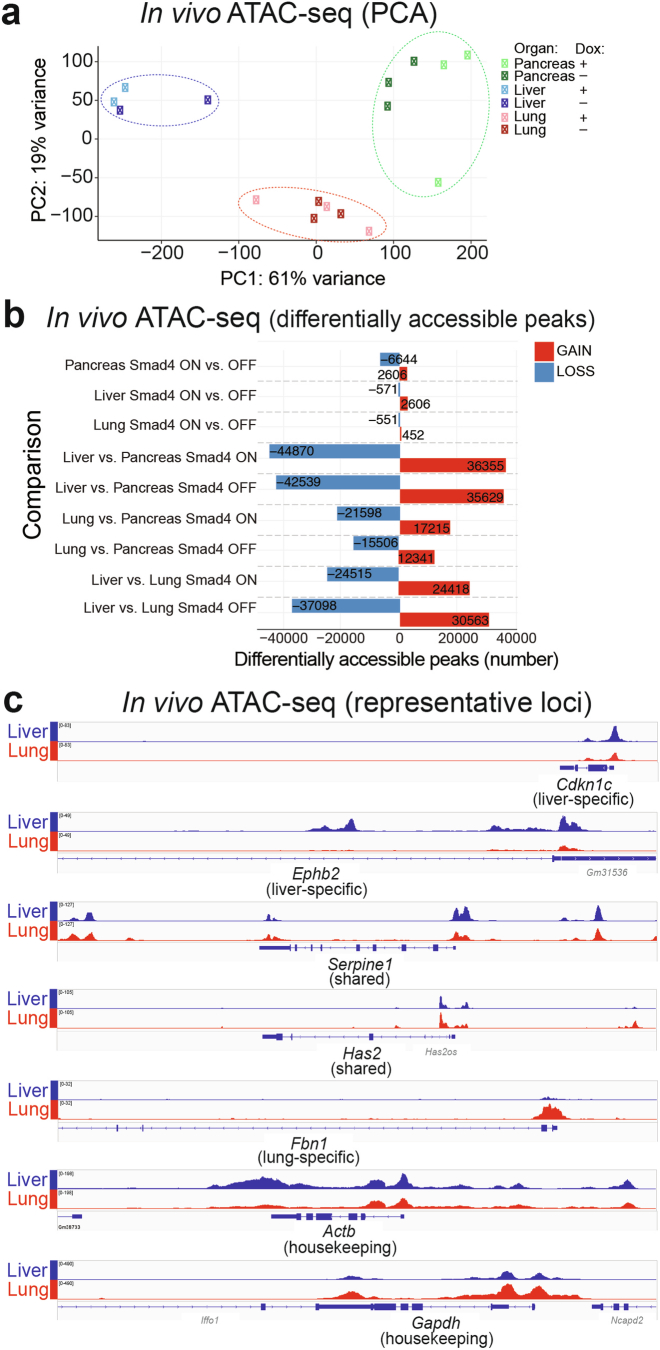
Analysis of *in vivo* ATAC-seq data. (**a**) Principal component analysis (PCA) of ATAC-seq data from tumor (mKate2^+^) cells isolated from the pancreas, liver, or lungs (based on peak normalization). Each sample corresponds to an independent mouse. Circled areas highlight separation based on organ. (**b**) Number of statistically significant differentially accessible *in vivo* ATAC-seq peaks. Exact numbers of gained/lost peaks are shown for each comparison (absolute fold change ≥1.5; FDR ≤0.1). (**c**) Representative *in vivo* ATAC-seq tracks at loci with liver-specific, lung-specific, or liver/lung-shared chromatin opening in KC-shSmad4 tumor cells (mKate2^+^GFP^+^). ‘Housekeeping’ genes are shown as a reference. Data are representative of 3 independent tumors. y-axis scale range is indicated per lane as normalized read counts. Source Data

Given the organ-specific transcriptional responses, we hypothesized that liver and lung metastases may harbor distinct chromatin states that afford different accessibility to SMAD4’s target genes. To test this, we performed ATAC-seq (assay for transposase-accessible chromatin using sequencing) on FACS-isolated tumor cells from the pancreas, liver and lungs ± *Smad4* restoration. Unsupervised clustering of differentially accessible peaks linked distinct chromatin states to tumors residing in different organs, whereas *Smad4* status itself had a limited impact (Fig. 5a, Extended Data Fig. 5a,b and Supplementary Table 3), consistent with the fact that it is not a ‘pioneer’ TF^27,32^. However, the differential chromatin accessibility affected SMAD4-dependent target genes, including its cytostatic and fibrogenic effectors highlighted earlier (Extended Data Fig. 5c). Furthermore, motif analysis of the liver-specific versus lung-specific ATAC-open regions revealed mostly distinct TF families, with the top unique predictions being KLF, HNF and ELF in the liver and RUNX, FOX and ETS in the lungs (Fig. 5b).

**Extended Data Fig. 6:**
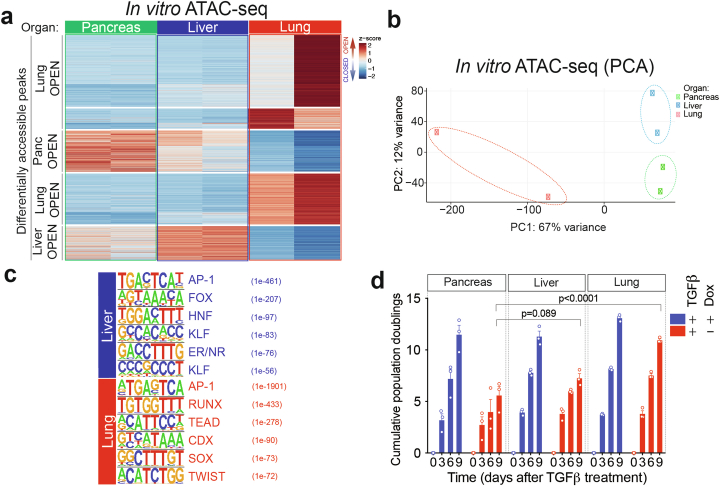
Analysis of *in vitro* ATAC-seq data and tumor-derived cell lines. (**a**) Heatmap of differentially accessible chromatin regions in Smad4 OFF tumor-derived cell lines ( + Dox) from the pancreas, liver, or lungs. Each column represents an independent cell line. A complete list of differentially accessible ATAC-seq peaks is provided in Supplementary Table 3. (**b**) Principal component analysis (PCA) of *in vitro* ATAC-seq data (based on peak normalization). Each sample corresponds to an independent cell line. Circled areas highlight separation based on organ site. (**c**) Top-scoring TF motifs identified by HOMER *de novo* motif analysis of *in vitro* ATAC-seq peaks enriched in liver vs. lung metastasis-derived cell lines. The numbers in parentheses indicate enrichment p-values. (**d**) Proliferation assays of KC-shSmad4 tumor-derived cell lines from the pancreas, liver, or lungs +/-Dox +TGFβ *in vitro* (n = 3 independent experiments per group). Statistical analyses were hypergeometric test (**c**), and one-way ANOVA (**d**). Source Data

In principle, these different chromatin states and corresponding TF activities could require ongoing exposure to factors in the organ microenvironment or be stably associated with distinct tumor cell subpopulations. To distinguish between these possibilities, we asked whether organ-biased differences in chromatin accessibility were maintained in vitro. We performed ATAC-seq on the Smad4-off primary tumor-derived cell lines used to generate liver and lung metastases and on matched cell lines derived from the respective metastatic sites. Remarkably, cell lines from the three different organs showed different chromatin accessibility profiles (Extended Data Fig. 6a,b). Motif analysis of the liver-specific versus lung-specific ATAC-open regions further uncovered distinct TF families that partially overlapped with their in vivo counterparts, including top enrichment for KLF and HNF motifs in the liver-derived cell lines and RUNX motifs in the lung-derived cell lines (Extended Data Fig. 6c). These data indicate that, while there are in vivo organ-enforced effects on the chromatin state of tumor cells, specific TF activities associated with distinct organ microenvironments and SMAD4 responses are heritable.

We then assessed the functional consequences of the inherited organ-biased cell states using in vitro and in vivo assays. The lung-derived but not liver-derived cells had a blunted cytostatic response to TGFβ in vitro relative to their matching primary tumor cells, indicating that the lung favors a cell-intrinsic state that is resistant to the tumor-suppressive effects of SMAD4 (Extended Data Fig. 6d). In addition, we performed in vivo ‘swap’ experiments where we used Smad4-off lung-derived cell lines in liver metastasis assays or Smad4-off liver-derived cell lines in lung metastasis assays, followed by *Smad4* restoration. The lung-into-liver swap abrogated SMAD4’s tumor-suppressive response in the liver, while the liver-into-lung swap impaired SMAD4’s tumor-promoting response in the lung, which was reflected in the unchanged tumor burden and fractions of Ki67^+^ and p57^+^ cells upon *Smad4* restoration in both organs (Fig. 5c–h). These data suggest that the heritable liver-associated and lung-associated epigenetic states have a critical role in defining the metastatic tumor’s response to TGFβ and SMAD4.

Collectively, these results show that dissemination to the liver versus lungs favors distinct chromatin states in tumor cells, whereby certain TF activities are stably inherited and associated with opposite dependence on *Smad4* inactivation.

### Early emergence of distinct metastasis-like chromatin states

**Fig. 6:**
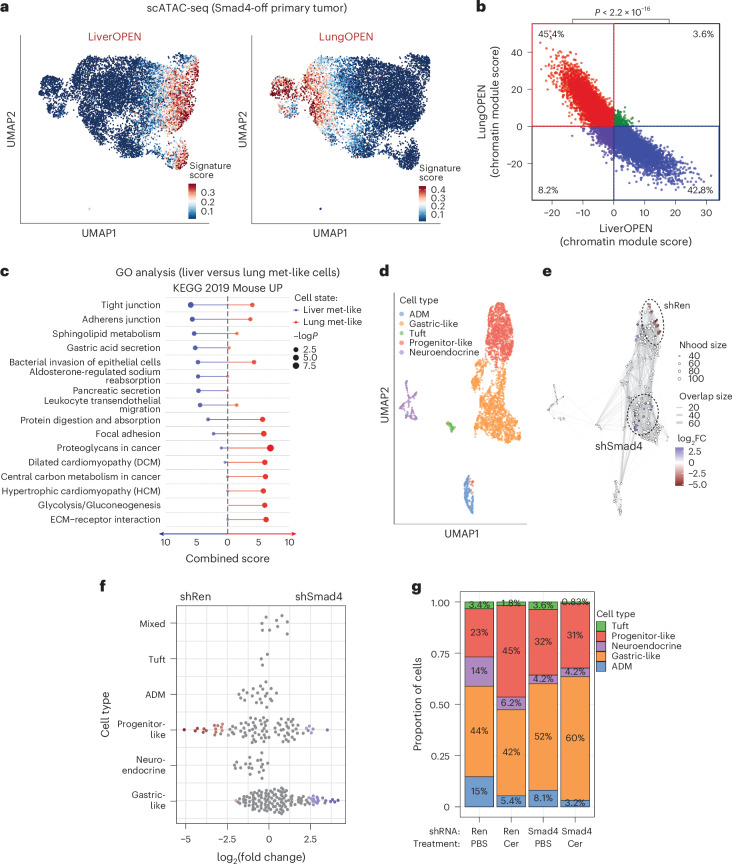
Liver and lung metastasis-like chromatin states emerge early in tumorigenesis. **a**, UMAP visualization of scATAC-seq profiles of KC-shSmad4 (+Dox) primary tumor cells (mKate2^+^). Signature scores based on liver-specific or lung-specific ATAC-open peaks from bulk ATAC-seq data are displayed in color per individual cell. Data represent three independent mice. **b**, Mutual exclusivity of liver-specific and lung-specific open chromatin signatures in primary tumors. The plot shows the distribution of cells from KC-shSmad4 (+Dox) primary tumors according to their enrichment of liver-specific (LiverOPEN) and lung-specific (LungOPEN) open chromatin signatures derived from bulk ATAC-seq (Methods). Each dot corresponds to an independent cell. The proportions of cells with liver-only (blue) and lung-only (red) signatures were compared to those with both liver and lung signatures (green) and those with neither signature (purple). **c**, GO analysis of upregulated genes in liver metastasis-like versus lung metastasis-like cell subpopulations of KC-shSmad4 primary tumors. Combined scores and *P* values for the top KEGG pathways are shown. Complete GO lists are provided in Supplementary Table 4. **d**, UMAP visualization of scRNA-seq profiles of KC-shRen and KC-shSmad4 (+Dox) premalignant pancreatic tissue (mKate2^+^). Cell type classification based on established gene signatures (Methods) are displayed in color per cell. Data represent four independent mice. **e**, Assessment of cell states enriched in shRen (red) versus shSmad4 (blue) conditions using Milo analysis (Methods). **f**, Distribution of premalignant pancreas cell neighborhoods in shRen or shSmad4 mice across transcriptional states. The *x* axis indicates the log_2_(fold change) in differential abundance of shRen (<0) and shSmad4 (>0). Each neighborhood was associated with a cell type if more than 80% of the cell states in the neighborhood belonged to the respective state; otherwise, it was annotated as ‘mixed’. **g**, Fractions of premalignant pancreas cells corresponding to different transcriptional states under the indicated conditions (*n* = 1 mouse per condition; Methods). PBS was used as the control. Cer, cerulein. Statistical analyses were conducted using a chi-squared test (**b**) or Fisher’s exact test with Benjamini–Hochberg adjustment (**c**). Source Data

Given the existence of heritable organ-associated epigenetic programs, we next asked whether they were already present in the primary tumor. To this end, we performed single-cell multiomics (scATAC-seq + scRNA-seq) on three independent KC-shSmad4 primary tumors and then mapped liver-enriched and lung-enriched open chromatin peaks identified in our bulk ATAC-seq on the single-cell space. This analysis identified distinct primary tumor cell subpopulations that harbored chromatin states resembling the states enriched in established liver and lung metastases (Fig. 6a,b).

To identify pathways associated with these two chromatin states, we performed GO analysis of the corresponding RNA-seq data. This analysis revealed that the liver versus lung metastasis-like states have distinct transcriptional programs that were previously linked to organ-specific metastasis. Liver metastasis-like cells showed a strong enrichment for tight junction genes (Fig. 6c and Supplementary Table 4), which facilitate interactions of tumor cells with hepatocytes to promote metastatic outgrowth in the liver^33^. By contrast, lung metastasis-like cells upregulated integrin and focal adhesion genes (Fig. 6c and Supplementary Table 4), known to promote metastatic outgrowth in the lungs^34^. Thus, liver and lung metastasis-like chromatin states can be found in distinct cell subpopulations of the primary tumor and are associated with different transcriptional programs that favor metastatic outgrowth at each respective organ site.

**Extended Data Fig. 7:**
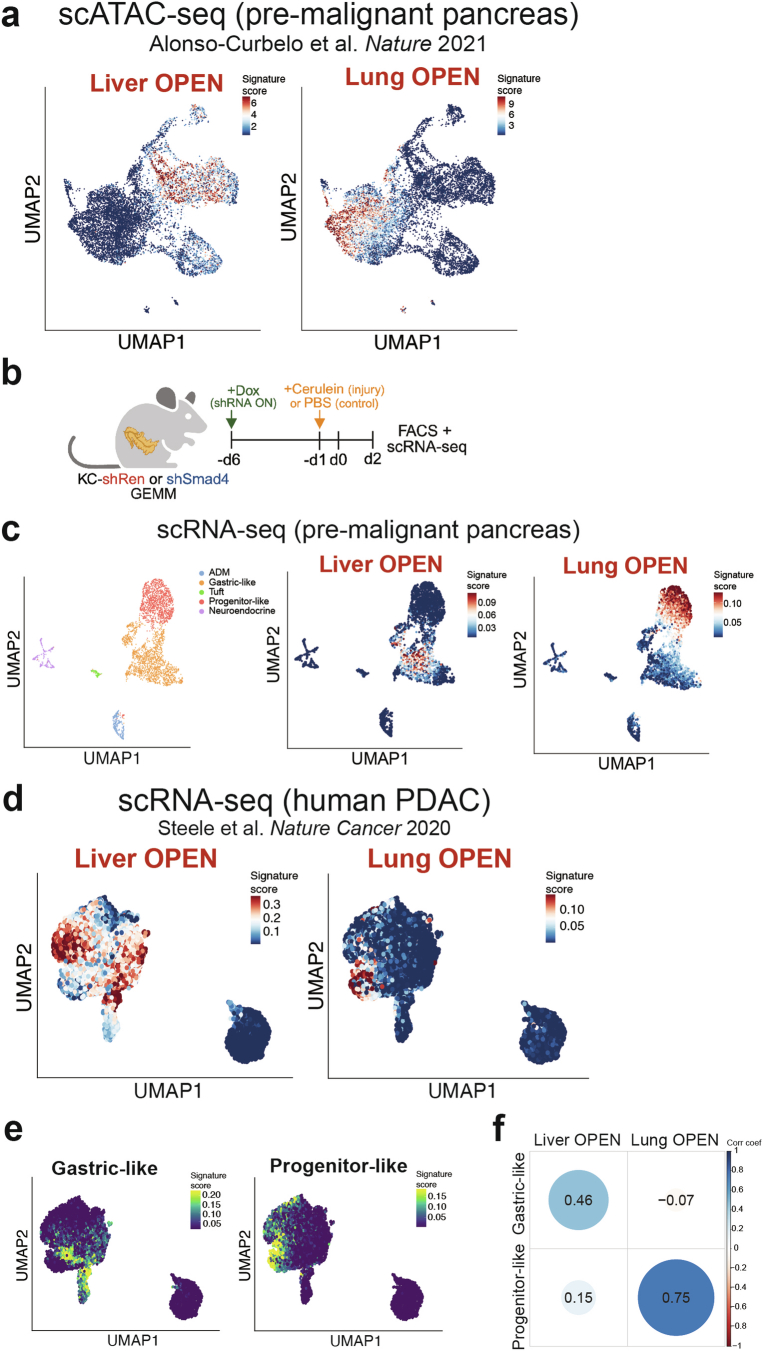
Analysis of liver and lung metastasis-like chromatin and transcriptional states in murine premalignant pancreas and human PDAC. (**a**) UMAP visualization of scATAC-seq profiles of *Kras*-mutant pancreatic epithelial cells (mKate2^+^) *+/-* cerulein injury (n = 1 mouse for each) from ref. ^35^ (see Methods for details). Signature scores based on liver- or lung-specific ATAC-open peaks from bulk ATAC-seq data are displayed in color per individual cell. (**b**) Schematic of scRNA-seq experiment in premalignant pancreas. Created in BioRender. Tsanov, K. (2025) https://BioRender.com/52xbslm. (**c**) (Left) UMAP visualization of scRNA-seq profiles of KC-shRen and KC-shSmad4 ( + Dox) pre-malignant pancreatic tissue (mKate2^+^) (identical to Fig. 6d). (Middle & Right) Signature scores based on liver- or lung-specific genes corresponding to organ-enriched ATAC-open peaks are displayed in color per individual cell. Data represent 4 independent mice. (**d**) UMAP visualization of scRNA-seq profiles of human primary PDAC tumor cells from ref. ^37^ (see Methods for details). Signature scores based on scRNA-seq profiles of mouse liver- or lung-specific ATAC-open cell populations (from the matching scATAC-seq multiomics data) are displayed in color per individual cell. (**e**) Gastric- and progenitor-like signature scores corresponding to panel (**d**). (**f**) Correlation analysis of liver/lung metastasis-like vs. gastric/progenitor-like transcriptional signatures. The size and color of each circle reflects correlation strength. Exact correlation scores are shown for each comparison. Statistical analysis by Pearson correlation. Source Data

We also probed scATAC-seq data obtained from premalignant pancreatic tissue (harboring only *Kras*^G12D^)^35^. Remarkably, this analysis identified similar chromatin states to those that occurred in advanced KC-shSmad4 primary tumors, implying that the organ-specific chromatin states arise even before cells acquire malignant potential (Extended Data Fig. 7a). To further explore the origin of these chromatin states, we then performed scRNA-seq on premalignant pancreata in our autochthonous model ± *Smad4* depletion and cerulein-induced pancreatitis, an environmental condition that facilitates PDAC development^35^ (Extended Data Fig. 7b). Analysis of these data also identified distinct subpopulations of premalignant cells that resembled liver or lung metastases, confirming at the transcriptional level our earlier conclusion that the two metastatic states begin to emerge even before malignancy (Extended Data Fig. 7c).

Next, we compared the liver and lung metastasis-like cell states seen in early neoplasia with established premalignant transcriptional signatures, including two distinct PDAC precursor cell types known as gastric-like and progenitor-like^36^. The two metastatic states showed opposite enrichment, whereby the liver-favored state was enriched in gastric-like cells, whereas the lung-favored state was enriched in progenitor-like cells (Extended Data Fig. 7c). Importantly, *Smad4* deficiency and tissue injury interacted to impact the distribution of these states. Specifically, cerulein treatment strongly promoted the progenitor-like state in the control shRNA-expressing pancreas, as previously reported^36^. By contrast, cerulein treatment promoted the gastric-like state in the sh*Smad4*-expressing pancreas (Fig. 6d–g). Thus, the liver and lung metastasis-like chromatin states are associated with distinct PDAC precursor cell types and are causally affected by *Smad4* and tissue injury.

Lastly, we asked whether the identified liver-biased and lung-biased metastatic states can be found in human PDAC. We leveraged the multiomic nature of our mouse single-cell data to generate matching RNA-seq signatures of the cell populations that were enriched for liver-specific versus lung-specific chromatin peaks in the ATAC-seq analysis. The resulting transcriptional signatures were then used to query scRNA-seq data from 16 human primary PDAC samples^37^. This analysis confirmed the existence of distinct cell subpopulations in the human primary tumors that resemble the organ-biased cell states in our mouse model and likewise show association with gastric-like versus progenitor-like cell types (Extended Data Fig. 7d–f). Overall, our data show that the liver and lungs favor metastatic cells harboring different chromatin states that begin to emerge in distinct subpopulations of the premalignant pancreas, are differentially favored by *Smad4* status and tissue injury, and are maintained in primary tumors.

### KLF4 and RUNX1 in liver-biased and lung-biased chromatin states

**Fig. 7:**
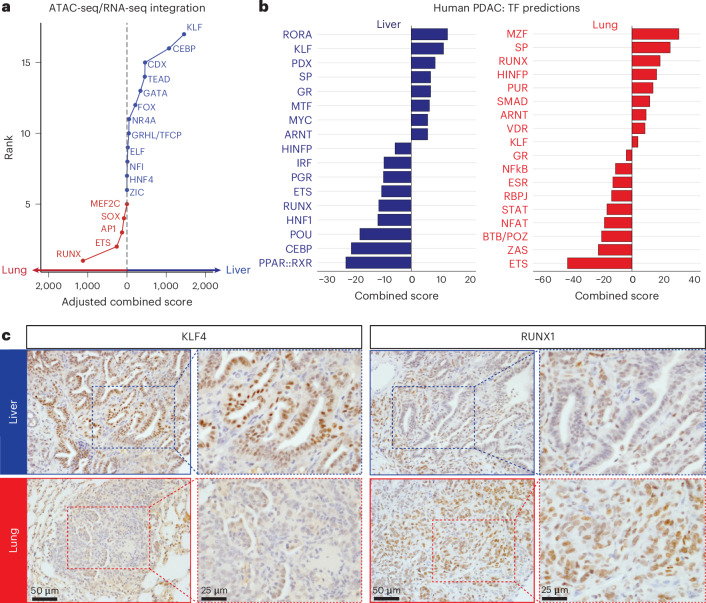
KLF4 and RUNX1 are associated with liver-biased and lung-biased chromatin states. **a**, ATAC-seq and RNA-seq combined scores for the indicated TF families in liver versus lung metastases. This metric infers the probability that a given TF family with a significantly enriched motif in the ATAC-open regions impacts SMAD4-induced gene expression changes on the basis of a consistent RNA-seq change in the Smad4 on versus off comparison (Methods). Top TF families scored using the HOMER de novo motif analysis in the liver versus lungs are shown. **b**, Combined scores for the indicated TF families in liver and lung metastases from PDAC patients^40^. This metric infers the activity of a given TF family on the basis of enrichment or depletion of its predicted target genes in the respective metastases versus primary tumors but not in the corresponding normal tissues (Methods). Top TFs scored using JASPAR or TRANSFAC position weight matrix data are shown. **c**, Representative IHC staining for KLF4 and RUNX1 in KC-shSmad4 (+Dox) liver or lung metastases. Right, higher-magnification views of the dashed areas to highlight tumor-specific nuclear signal. Data are representative of 20 metastases from four independent mice. Source Data

To determine which TFs may cooperate with SMAD4 to impact organ-specific gene expression, we integrated our ATAC-seq and RNA-seq datasets to identify TF families (1) whose motifs were enriched in the differentially accessible chromatin regions and (2) whose predicted targets were upregulated upon *Smad4* restoration. This analysis pinpointed the KLF and RUNX families of pioneer TFs^32,38,39^ as candidate drivers in the liver and lungs, respectively (Fig. 7a). To assess which TF families are of highest relevance to human PDAC, we then used transcriptomic data from metastatic human PDAC^40^ to impute differential TF activity in liver or lung metastases on the basis of enrichment or depletion of a given TF’s target genes relative to primary tumors (Methods). Corroborating our mouse data, KLF targets were enriched in liver metastases, whereas RUNX targets were depleted in liver and enriched in lung metastases (Fig. 7b). These results nominate the KLF and RUNX TF families as organ-specific determinants of chromatin-directed transcriptional programs responsive to SMAD4.

**Extended Data Fig. 8:**
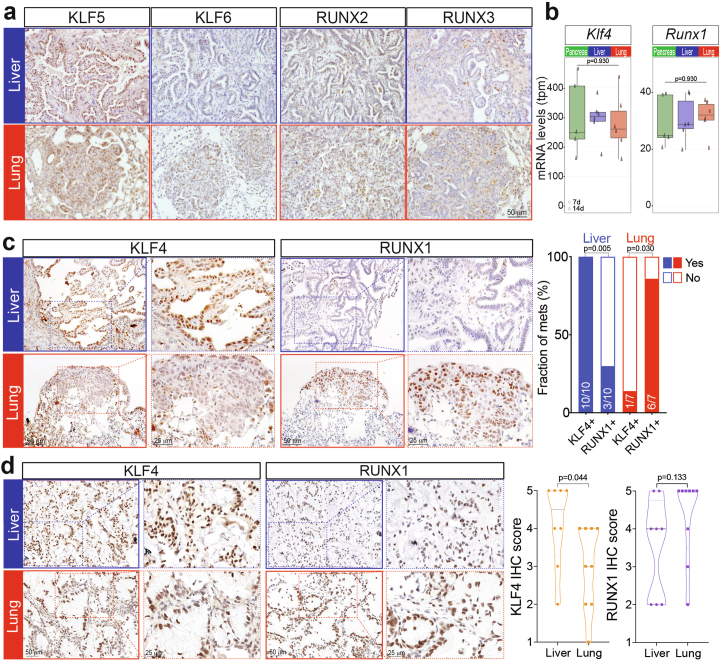
Analysis of KLF and RUNX TF expression in liver and lung metastases from murine transplant models, GEMMs, and PDAC patients. (**a**) Representative IHC staining for the indicated TFs in KC-shSmad4 ( + Dox) liver or lung metastases. Data are representative of 20 metastases from 4 independent mice. (**b**) Normalized *Klf4* and *Runx1* mRNA levels (RNA-seq data; tpm = transcripts per million) in KC-shSmad4 tumor cells from the pancreas, liver, or lungs ( + Dox for 7 or 14 days) (n = 5, 6, 6, 5, 6 and 6 independent mice for groups shown, left to right). Box plots represent median +/- upper/lower quartile (box) +/-1.5 x IQR between the first and third quartile. (**c**) Representative IHC staining for KLF4 and RUNX1 in KC-shSmad4 ( + Dox) GEMM liver or lung metastases. Higher magnifications of dashed areas are shown on the right to highlight tumor-specific nuclear signal. Quantification of the fraction of KLF4+ or RUNX1+ metastases is shown on the right (n = 10 and 7 independent tumors from 5 and 4 mice for liver and lung, respectively). (**d**) Representative IHC staining for KLF4 and RUNX1 in liver or lung metastases from PDAC patients. Higher magnifications of dashed areas are shown on the right to highlight tumor-specific nuclear signal. Quantification of the KLF4 or RUNX1 IHC signal is shown on the right (n = 8 and 9 independent tumors from 7 and 8 patients for liver and lung, respectively). Statistical analyses were one-way ANOVA (**b**), chi-squared test with Yates correction (**c**), and two-sided Mann-Whitney *U* test (**d**). Source Data

The KLF and RUNX families contain multiple TFs that have been implicated in PDAC biology. Among them, KLF4 and KLF5 are both enforcers of pancreatic epithelial identify^11,41,42^, whereby KLF5 silencing by TGFβ compromises PDAC cell survival^11^. Interestingly, KLF4 can exhibit protumorigenic or antitumorigenic effects in PDAC depending on context, despite binding similar DNA motifs to KLF5 (refs. ^11,42,43^). On the other hand, RUNX1 and RUNX3 have been implicated as drivers of invasion and metastasis and as potential genetic dependencies in PDAC^16,44,45^. To define which specific TFs are most likely to underlie the observed organ-specific phenotypes, we performed IHC staining for each of these factors in KC-shSmad4 metastases. KLF4 exhibited strong specificity for liver metastases and RUNX1 for lung metastases; at the same time, KLF5, KLF6 and RUNX2 presented signal in both organs, albeit to a different extent, and RUNX3 did not show a tumor-specific signal but rather stained stromal cells in our model (Fig. 7c and Extended Data Fig. 8a). Interestingly, the mRNA levels of *Klf4* and *Runx1* did not differ significantly between liver and lung metastases, suggesting post-transcriptional regulation of these TFs (Extended Data Fig. 8b).

Importantly, the organ preference of KLF4 and RUNX1 for liver and lung metastases was confirmed in the autochthonous KC-shSmad4 model, where ten of ten liver metastases were KLF4^+^ (versus three of ten RUNX1^+^) and six of seven lung metastases were RUNX1^+^ (versus one of seven KLF4^+^) by IHC (Extended Data Fig. 8c). IHC analysis of metastatic tissue from 17 PDAC patients corroborated higher KLF4 expression in liver metastases, albeit to a lesser degree than in the mouse models (Extended Data Fig. 8d). RUNX1 showed a trend toward higher signal in lung metastases but did not reach statistical significance at the analyzed number of samples (Extended Data Fig. 8d), implying that KLF4 status has a dominant role in the human setting.

**Extended Data Fig. 9:**
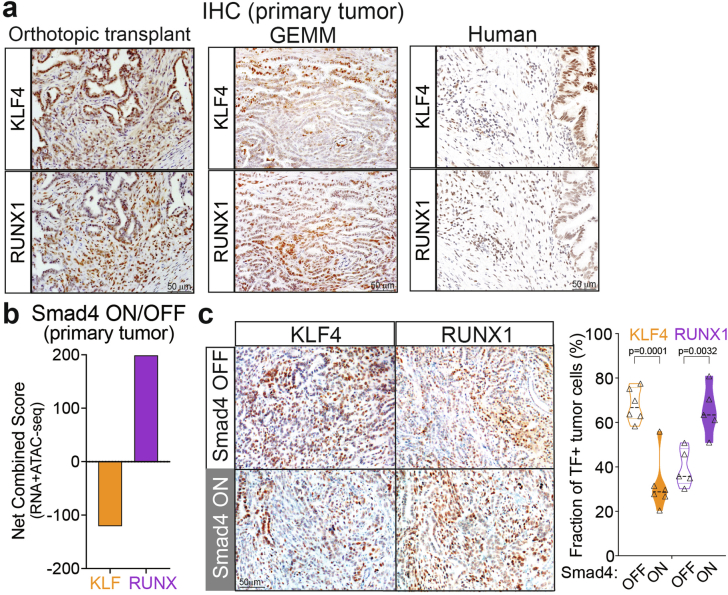
Analysis of KLF4 and RUNX1 expression in murine and human primary PDAC tumors. (**a**) Representative IHC staining for KLF4 and RUNX1 in serial sections of a primary tumor from the KC-shSmad4 ( + Dox) transplant model (left), autochthonous model (middle), and human PDAC patient (right). Data are representative of 4 (transplant model) and 6 (autochthonous model) independent mice and 8 patients. (**b**) Net change in ATAC-RNA combined scores for the KLF and RUNX TF families in Smad4 ON vs. OFF primary tumors at day 14. This metric infers the probability that a given TF family is impacted by *Smad4* restoration, based on a combined change in ATAC-seq motif accessibility and RNA-seq levels of its predicted target genes (see Methods for details). (**c**) Representative IHC staining for KLF4 or RUNX1 in KC-shSmad4 primary tumors +/-*Smad4* restoration. Quantifications are shown on the right (n = 6, 6, 5 and 5 independent tumors per group, respectively). Analysis was performed 14 days after Dox withdrawal. Statistical analysis was unpaired two-sided t-test. Source Data

Interestingly, IHC staining for KLF4 and RUNX1 in primary tumors was heterogeneous across the transplantation and autochthonous mouse models and patients, indicating broad expression of both TFs in apparently nonoverlapping subsets of cells (Extended Data Fig. 9a). These data implied that the chromatin states associated with KLF and RUNX—and their opposite responsiveness to SMAD4—are present in subpopulations within the primary tumor, as suggested earlier by our scATAC-seq and scRNA-seq analyses. To further address this, we integrated our ATAC-seq and RNA-seq data from primary tumors ± *Smad4* restoration to infer KLF and RUNX TF activities. The latter were defined by combining metrics of chromatin accessibility at the respective TF motif with transcriptional changes in the TF’s target genes (Methods). This analysis revealed that *Smad4* restoration caused a decrease in inferred KLF activity and an increase in inferred RUNX activity in the pancreas (Extended Data Fig. 9b). In agreement, IHC staining revealed a reduced fraction of KLF4^+^ cells and an increased proportion of RUNX1^+^ cells after 14 days of *Smad4* restoration (Extended Data Fig. 9c). These data support the concept that the KLF and RUNX-associated cell states pre-exist in the primary tumor, whereby SMAD4 antagonizes KLF activity while cooperating with RUNX activity.

Overall, our refined analysis nominates KLF4 and RUNX1 as particular TFs that are likely to mediate organ-specific chromatin opening and thereby differential dependence on *Smad4* inactivation in liver versus lung metastases.

### Organ-specific interplay of *Klf4* and *Runx1* with *Smad4*

**Extended Data Fig. 10:**
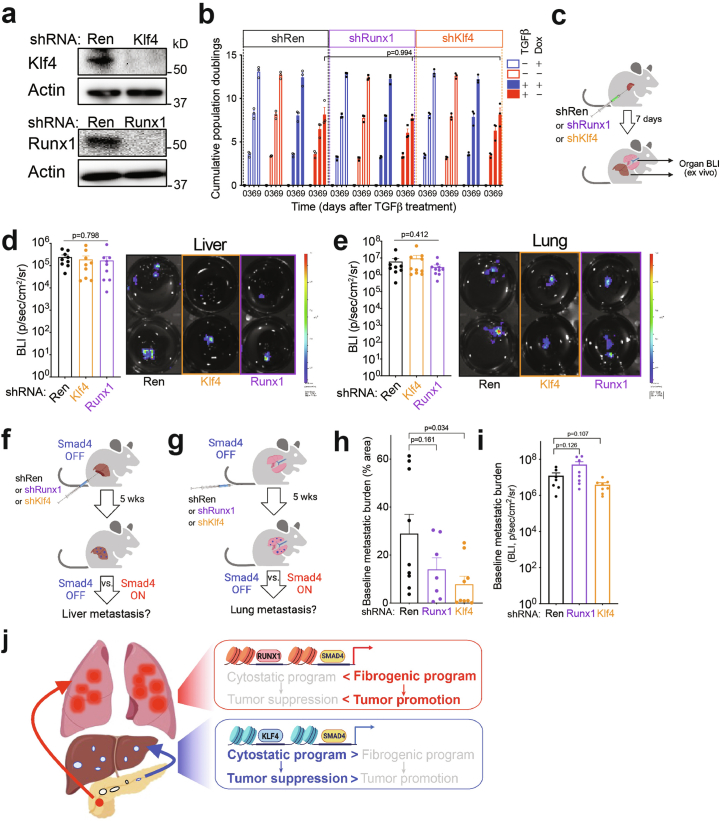
Analysis of *Klf4* and *Runx1* depletions *in vitro* and *in vivo.* (**a**) Western blot analysis of shRNA efficiency. Actin was used as a loading control. (**b**) Proliferation assays of KC-shSmad4 cell lines harboring the indicated stable shRNAs +/-Dox and +/-TGFβ *in vitro* (mean + SEM; n = 3 independent experiments per group). (**c**) Schematic of experiments to assess early metastatic colonization of KC-shSmad4 cells with TF knockdowns. Created in BioRender. Tsanov, K. (2025) https://BioRender.com/ufvotxg. (**d-e**) Bioluminescent analysis of livers (**d**) and lungs (**e**) harvested 7 days after intracardiac injection of cells expressing the indicated shRNAs. Quantification is shown on the left and representative bioluminescent images on the right (mean + SEM; n = 9, 10 and 10 independent mice per organ for groups shown, left to right). (**f-g**) Schematic of intrasplenic (**f**) and tail vein (**g**) experiments with KC-shSmad4 cells with TF knockdowns. Created in BioRender. Tsanov, K. (2025) https://BioRender.com/ur1sduv and https://BioRender.com/hxar0yr. (**h**) Quantifications of baseline liver metastasis burden (% tumor area) after intrasplenic injections of KC-shSmad4 cells under continuous Dox administration (Smad4-OFF) (mean + SEM; n = 9, 7 and 9 independent mice per group, respectively). Livers were harvested 64-66 days after injection. (**i**) Quantifications of baseline lung metastasis burden (bioluminescent signal) after tail vein injections of KC-shSmad4 cells under continuous Dox administration (Smad4-OFF) (mean + SEM; n = 7, 8 and 8 independent mice per group, respectively). Lungs were harvested 57 days after injection. (**j**) Model of the organ-specific interplay between SMAD4 and KLF4/RUNX1-associated chromatin states. In the liver, active KLF4 allows SMAD4 to act at genes that promote cytostasis, thus necessitating the inactivation of *Smad4*. In the lungs, more speculatively, active RUNX1 allows SMAD4 to act at pro-fibrotic genes unopposed by its cytostatic program. Partially created in BioRender. Tsanov, K. (2025) https://BioRender.com/74y5yim. Source Data

To functionally interrogate the roles of *Klf4* and *Runx1* and their interplay with *Smad4* in liver and lung metastases, we used stable shRNA-based knockdown to deplete *Klf4* or *Runx1* (or an shRNA to target *Renilla* luciferase as a neutral control) in KC-shSmad4 cells. While both sh*Klf4* and sh*Runx1* achieved a potent reduction in the respective proteins (Extended Data Fig. 10a), the corresponding cell lines had unaltered basal proliferation and response to TGFβ upon *Smad4* restoration in vitro in comparison to the sh*Ren* control (Extended Data Fig. 10b). We then assessed the impact of these knockdowns on early metastatic colonization by intracardiac injection of these cells in mice on Dox (Smad4 off), followed by organ isolation and bioluminescence analysis 7 days later (Extended Data Fig. 10c). All conditions displayed a similar signal in the livers and the lungs, indicating that *Klf4* and *Runx1* do not affect seeding and early colonization of these organs (Extended Data Fig. 10d,e). In line with our scRNA-seq data showing enrichment of pathways that promote tumor outgrowth in the liver and lungs (Fig. 6c), these results imply that the KLF4^+^ and RUNX1^+^ cell states associated with liver and lung metastases are selected during expansion at the respective metastatic site.

**Fig. 8:**
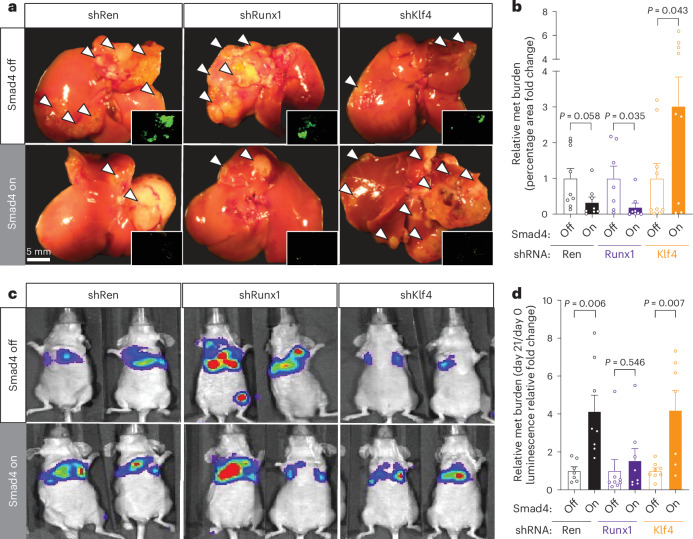
*Klf4* and *Runx1* depletions antagonize *Smad4* in an organ-specific manner. **a**, Representative macroscopic images of tumor-bearing livers ± *Smad4* restoration on the background of the indicated stable shRNAs. Arrowheads highlight metastatic tumors. Insets, shSmad4-linked GFP reporter. Data are representative of the number of independent mice per group specified in **b**. For shRen, the same cohort as shown in Fig. 4 was used. **b**, Quantifications of metastasis burden (fold change of percentage tumor area) on day 30 after Dox withdrawal (mean ± s.e.m.; *n* = 9, 8, 7, 8, 9 and 9 independent mice per group, respectively). For shRen, the same cohort as shown in Fig. 4 was used. **c**, Representative bioluminescence images of tumor-bearing lungs ± *Smad4* restoration on the background of the indicated stable shRNAs. Data are representative of the number of independent mice per group specified in **d**. **d**, Quantifications of metastasis burden (fold change of bioluminescence signal) on day 21 after Dox withdrawal (mean ± s.e.m.; *n* = 7, 8, 8, 8, 8 and 7 independent mice per group, respectively). Statistical analysis was conducted using an unpaired two-sided *t*-test (**b**,**d**). Source Data

To assess the TF interplay with *Smad4* during metastatic outgrowth, we then generated liver or lung metastases through intrasplenic or tail-vein injection of the shRNA-expressing cells in mice on Dox (Smad4 off), as described earlier (Extended Data Fig. 10f,g). Consistent with a general role for *Klf4* in supporting a liver-metastatic cell state, its depletion reduced baseline tumor burden in the liver; meanwhile, *Runx1* depletion produced a smaller reduction in liver metastasis burden that did not reach statistical significance (Extended Data Fig. 10h). Upon *Smad4* reactivation, liver metastases expressing sh*Ren* and sh*Runx1* continued to show a reduction in tumor burden consistent with SMAD4-mediated tumor suppression, whereas those expressing sh*Klf4* now exhibited a threefold increase in metastasis burden, akin to the behavior of lung metastases (Fig. 8a,b). By contrast, *Klf4* depletion in lung metastases had no effect on the tumor-promoting effects of SMAD4, whereas *Runx1* depletion blunted this effect (Fig. 8c,d and Extended Data Fig. 10i). Thus, *Klf4* and *Runx1* depletions are sufficient to reverse and blunt *Smad4* function in liver and lung metastases, respectively, which demonstrates that KLF4 facilitates *Smad4*’s tumor-suppressive activity in the liver and RUNX1 contributes to *Smad4*’s tumor-promoting activity in the lungs (Extended Data Fig. 10j).

## Discussion

Our results demonstrate how driver gene mutations that are important for tumor initiation can show opposite requirements for maintenance at metastatic sites and place the paradigmatic duality of TGFβ signaling in an anatomic context. While it has been established that *SMAD4* mutation can switch TGFβ’s activity from tumor-suppressive to tumor-promoting^12^, we show here that this switch can also be mediated by the organ location of otherwise isogenic tumors. *SMAD4* loss itself, thus, does not universally favor tumor growth because its inactivation appears to be a liability rather than an advantage for lung metastases, at least in the clinically relevant setting of preseeded metastases modeled in our study. Hence, our data provide a possible reason for the unusually low rates of *SMAD4* inactivation in isolated lung metastases of PDAC patients^25,26,46^.

Mechanistically, the involvement of KLF and RUNX factors is consistent with both the operational logic of TGFβ signaling—whose contextual effects are often defined by interplay with such developmental TFs^27^—and the reported functions of these TFs in PDAC and other settings. In particular, certain KLF factors promote the epithelial cell fate^41^, which in turn is known to be essential for colonization of the liver^47–49^, whereas RUNX proteins facilitate extracellular matrix remodeling that can create a prometastatic fibrogenic microenvironment in the lungs when unopposed by a tumor-suppressive program^50^. Future studies will determine the extent to which organ-specific effects apply to other common cancer drivers, many of which also enhance metastatic proclivity; such drivers include missense mutations of the *TP53* tumor suppressor gene^51^, gains or amplifications at the mutant *KRAS*^22,52^ and *MYC*^53^ loci or deletions at the *CDKN2A* locus^21^.

Our results also add to the growing appreciation that tissue context can influence the output of gene mutations in cancer, for example, as illustrated by the differential susceptibility of cells from particular tissues or tissue locations to certain oncogenic events^54–56^. Our findings extend this concept to metastasis by showing how organ site can have a profound impact on a single driver mutation in a tumor from the same tissue of origin. As an underlying mechanism, we show that such mutations synergize with or antagonize distinct chromatin states that emerge remarkably early during tumorigenesis, in line with recent evidence that prometastatic epigenetic programs can arise even in premalignant tissues^35,50^ and metastatic organotropism can be predicted from transcriptional signatures in primary PDAC tumors^57^. Our data further relate these states to distinct PDAC precursors, raising the possibility that they may emerge in different cells of origin, which warrants future studies that incorporate cell-type-specific lineage-tracing tools. Importantly, we find that these distinct chromatin states are favored by different metastatic sites and can influence the expression of key target genes such as the cell-cycle inhibitor p57. Additional work will define the contribution of immune and other factors in the organ microenvironment that likely influence this gene–chromatin interplay^57^. Regardless, when extended to precision oncology, our results draw attention to potentially divergent responses to therapy based on the tumor’s organ of residence. As such, they are in line with clinical observations of organ heterogeneity in therapy response^58–60^ and invite consideration of organ-specific therapies for tumors driven by mutations that show such dependence on metastatic site.

## Methods

### Ethical regulations

The research performed in this study complied with all relevant ethical regulations. All mouse experiments were approved by the Memorial Sloan Kettering Cancer Center (MSKCC) Institutional Animal Care and Use Committee (IACUC) (protocol 11-06-018). Archival human specimens were procured under a biospecimen research protocol approved by the MSKCC Institutional Review Board (protocol 22-159). All participants provided preprocedure written informed consent.

### Animals and in vivo procedures

#### Animal care

Mice were maintained under pathogen-free conditions, housed on a 12-h light–dark cycle under ambient temperature of 18–24 °C and 40–60% humidity. Food and water were provided ad libitum. GEMMs were generated in house. *Foxn1*^*nu*^ (athymic nude) mice used for transplants were purchased from Envigo or The Jackson Laboratory. In all experiments, tumors did not exceed a maximum volume corresponding to 10% of the mouse’s body weight (typically 1 cm^3^), as per IACUC guidelines.

#### GEMMs

*Ptf1a*^*Cre*/+^;*LSL-Kras*^G12D/+^;*Rosa26*^*LSL-rtTA3-IRES-mKate2*/+^(*RIK*);*Col1a1*^*shRNA-Homing-Cassette*/+^(*CHC*) male embryonic stem cells (ES cells (C57BL/6J;129 mixed background) were targeted with two independent GFP-linked Smad4 shRNAs (shSmad4.591, CAAAGATGAATTGGATTCTTT; shSmad4.1599, ACAGTTGGAATGTAAAGGTGA) cloned into miR30-based targeting constructs, as previously described^61^. The KC-shRen control ES cell clone was previously reported^61^. Mice were generated by eight-cell or blastocyst injection of ES cells and shRNAs were induced by treatment of the resulting mice with Dox (625 mg kg^−1^; Envigo) in the chow starting at 5–6 weeks of age. To examine the effects of tissue injury and *Smad4* depletion on the premalignant pancreas, 8-week-old KC-shRen and KC-shSmad4 mice were treated with cerulein (80 μg kg^−1^, intraperitoneally; Bachem) or phosphate-buffered saline (PBS) by 8 hourly injections on two consecutive days. The identity of the ES cells and corresponding mice was authenticated by genomic PCR using a common Col1a1 primer paired with an shRNA-specific primer (Supplementary Table 5), all yielding ~250-bp products.

#### Orthotopic transplantation assays

Mouse hosts were placed on Dox chow 5–7 days before transplantation. Mice were anesthetized and a survival surgery was performed to expose the pancreas. A total of 1 × 10^5^ tumor-derived cells were resuspended in 25 μl of 1:1 DMEM (Gibco) and Matrigel (Corning) and injected in the tail of the pancreas of each mouse. Tumor engraftment and progression were monitored by palpation and ultrasound imaging. Where applicable, Dox withdrawal was performed 4 weeks after injection (corresponding to a 3–5-mm tumor diameter) by switching the food source to regular chow. At the experimental endpoint, primary tumors, livers and lungs were dissected and imaged under a dissection microscope for brightfield, mKate and GFP fluorescence (Nikon SMZ1500 with NIS-Element version 3.0 software). Liver and lung metastasis burden was measured by calculating the percentage tumor area (mKate^+^) as a fraction of overall organ area. Mice were killed upon reaching experimental or humane endpoints according to IACUC guidelines. All mice used were 6–8-week-old *Foxn1*^*nu*^ females.

#### Experimental metastasis assays

For liver metastasis assays, mice were anesthetized and a survival surgery was performed to expose the spleen. A total of 4 × 10^5^ tumor-derived cells resuspended in 20 μl of PBS were injected in the splenic parenchyma of each mouse, followed by removal of the spleen and cauterization (splenectomy). Tumor engraftment and progression were monitored by palpation and ultrasound imaging. At experimental endpoint, livers were dissected and imaged under a dissection microscope for brightfield, mKate and GFP fluorescence (Nikon SMZ1500 with NIS-Element version 3.0 software). Liver metastasis burden was measured by calculating the percentage tumor area (mKate^+^) as a fraction of overall organ area. For lung metastasis assays, mice were temporarily restrained and 2.5 × 10^5^ tumor-derived cells resuspended in 250 μl of PBS were injected in the tail vein of each mouse. Bioluminescence imaging was used to monitor tumor engraftment and progression and measure tumor burden. Where applicable, Dox withdrawal was performed 5 weeks (intrasplenic) or 6 weeks (tail vein) after injection (unless noted otherwise) by switching the food source to regular chow. Mice that developed tumors outside of the respective target organs were excluded from the analysis. Mice were killed upon reaching experimental or humane endpoints according to IACUC guidelines. All mice used were 6–8-week-old *Foxn1*^*nu*^ females.

#### Animal imaging

For ultrasound, mice were anesthetized and high-contrast ultrasound imaging was performed on a Vevo 2100 System with a MS250 13–24-MHz scanhead (VisualSonics). Images were acquired and tumor volume was measured using the Vevo LAB software (version 3.2; VisualSonics). For bioluminescence, mice were injected with luciferin (5 mg per mouse, intraperitoneally or retro-orbitally; Gold Technologies), anesthetized for 10 min and then imaged on a IVIS Spectrum imager (Perkin Elmer). Images were acquired and bioluminescence signal was measured using the Living Image software (version 4.5; Perkin Elmer).

#### Analysis of early metastatic colonization

A total of 1 × 10^5^ cells were resuspended in 100 μl of PBS and injected into the right cardiac ventricle of anesthetized mice with a 26G-needle syringe. Successful inoculation of luciferase-transduced cells was verified by bioluminescence imaging immediately after injection. After 7 days, mice were injected with luciferin (5 mg per mouse, retro-orbitally; Gold Technologies) and killed; their livers and lungs were isolated in PBS and immediately imaged ex vivo using the IVIS Spectrum imager and Living Image software (version 4.5; Perkin Elmer).

### Histological, IHC and immunofluorescence (IF) analyses

Tissues were fixed in 10% neutral buffered formalin overnight (Fisher Scientific, 22-050-105), paraffin-embedded and sectioned at 5-μm thickness. Hematoxylin and eosin (H&E) staining was performed using standard protocols. For immunostaining, slides were incubated at 55 °C for 30 min, deparaffinized and rehydrated. Antigen retrieval was performed using citrate buffer (Vector Laboratories, H-3300) in a pressure cooker for 25 min. Sections were treated with 3% H_2_O_2_ for 10 min and washed in deionized water (for IHC only), then washed in PBS and blocked in 5% BSA in PBS with 0.1% Triton X-100. Primary antibody incubation was performed at 4 °C overnight in blocking buffer. Primary antibodies were to SMAD4 (clone EP618Y, Millipore, 04-1033; 1:200, IHC), pSMAD2 (clone 138D4, Cell Signaling, 3108; 1:100, IHC), p57 (Atlas Antibodies, HPA002924; 1:500, IHC), KLF4 (Abcepta, AM2725A; 1:100, IHC), KLF5 (Abcam, ab137676; 1:500, IHC), KLF6 (Abcam, ab241385; 1:1,000, IHC), RUNX1 (clone D4A6, Cell Signaling, 8529; 1:500, IHC), RUNX2 (clone D1L7F, Cell Signaling, 12556; 1:500, IHC), RUNX3 (clone 2B3, Life Technologies, MA5-17169; 1:500, IHC), mKate2 (Evrogen, AB233; 1:1,000, IF), Ki67 (clone B56, BD Pharmingen, 550609; 1:200, IF) and α-SMA (clone 1A4, Sigma, A2547; 1:1,000, IF).

For IHC, horseradish peroxidase (HRP)-conjugated secondary antibodies (ImmPRESS kits, Vector Laboratories, MP7401 and #MP2400) were applied for 30–60 min at room temperature, followed by incubation with DAB substrate (ImmPACT kit, Vector Laboratories, SK-4105). Tissues were then stained with hematoxylin, dehydrated and mounted using Permount (Fisher Scientific, SP15-100). For IF, secondary Alexa Fluor 488 (A-21202) or 594 (A-21207) dye-conjugated antibodies (Life Technologies, 1:500) were applied for 60 min at room temperature. Tissues were then stained with DAPI and mounted using Prolong Gold (Invitrogen, P36930).

Image acquisition was performed on a Zeiss AxioImager microscope equipped with an ORCA/ER charge-coupled device camera (Hamamatsu Photonics) and ZEN software (version 3.3; Zeiss). For pSMAD2 and p57 quantifications, the number of pSMAD2^+^ or p57^+^ cells per randomly chosen IHC-stained ×40 fields of view in a tumor region was manually counted. For Ki67 and α-SMA quantification, random ×20 fields of view costained for mKate2 were analyzed. mKate2+ areas were selected and then (1) the corresponding Ki67^+^ cells were counted and calculated as a percentage of DAPI^+^ cells or (2) the corresponding α-SMA^+^ area was measured and calculated as a percentage of the mKate2^+^ area. Image analyses were performed using ImageJ/FIJI (National Institutes of Health (NIH)).

### Cloning

ES cell-targeting plasmids were generated as described above. The firefly luciferase reporter plasmid (pMSCV-Luc2-Blast) was generated by subcloning Luc2 from the pCDH-EF1-Luc2-P2A-tdTomato plasmid into the pMSCV-Blasticidin retroviral vector at the BglII–HpaI restriction sites with the inclusion of a Kozak sequence (GCCACC) upstream of the ATG start codon, using standard protocols. Constitutive *Renilla*, *Cdkn1c* (p57), *Klf4* and *Runx1* shRNAs were cloned in the pMSCV-mirE-SV40-Neomycin-BFP retroviral vector^62^ at the XhoI–EcoRI restriction sites, using standard protocols. pCDH-EF1-Luc2-P2A-tdTomato was a gift from K. Oka (Addgene, 72486). pMSCV-Blasticidin was a gift from D. Mu (Addgene, 75085)^63^. All plasmids were authenticated by test digestion and Sanger sequencing. The following shRNA sequences were used:

- *Ren*, 5′-GCAGGAATTATAATGCTTATC-3′
- p57 (*Cdkn1c*), 5′-TGGTAATAATCAATAACCCAG-3′
- *Klf4*, 5′-TATAAAAATAGACAATCAGCA-3′
- *Runx1*, 5′-AAATCAGAAGCATTCACAGTT-3′

### Cell culture

All cells were maintained in a humidified incubator at 37 °C with 5% CO_2_.

#### Primary cell line derivation

Cell lines were generated from tumor-bearing pancreata, livers or lungs of KC-shSmad4 or KC-shRen mice. Tumors were dissected, chopped with razor blades and digested with 1 mg ml^−1^ collagenase V (Sigma-Aldrich) diluted in HBSS for 30–60 min, followed by 0.25% trypsin for 5–10 min. Digested tissues were washed with complete DMEM (DMEM, 10% FBS (Gibco) and 1× penicillin–streptomycin), passed through a 100-μm filter and cultured in complete DMEM on collagen-coated plates (0.1 mg ml^−1^ PureCol, Advanced Biomatrix) supplemented with 1 μg ml^−1^ Dox at 37 °C. Cells were passaged at least five times to eliminate any nontumor cells before using them in experiments. Primary cultures were authenticated by flow cytometry of engineered fluorescence alleles. All cultures were tested negative for *Mycoplasma*.

#### Virus generation and transduction

For stable transduction of firefly luciferase and constitutive shRNA constructs, VSV-G pseudotyped retroviral supernatants were generated from transduced Phoenix-GP packaging cells and infections were performed as described elsewhere^21^. Infected cells were selected with 10 μg ml^−1^ Blasticidin for 5 days or 800 μg ml^−1^ G418 for 7 days, depending on the selection marker. Luciferase expression was confirmed by in vitro and in vivo bioluminescence. Knockdowns were confirmed by western blot using standard procedures and antibodies to SMAD4 (clone B-8, Santa Cruz, sc-7966; 1:500), GFP (clone D5.1, Cell Signaling, 2956; 1:1,000), p57 (Atlas, HPA002924; 1:1,000), KLF4 (Abcepta, AM2725A; 1:1,000), RUNX1 (Cell Signaling, 8529; 1:1,000) and actin–HRP (clone AC-15, Sigma, A3854; 1:20,000).

#### Proliferation assays

For ‘+Dox’ conditions, cells were cultured under continuous Dox treatment (1 μg ml^−1^). For ‘−Dox’ conditions, cells were cultured in the absence of Dox for 5 days before the start of the experiment (day 0). On day 0, 12.5 × 10^3^ cells were plated on collagen-coated 24-well plates, in the presence or absence of 100 pM recombinant mouse TGFβ1 (R&D Systems, 7666-MB-005). Cells were counted every 3 days (that is, on days 3, 6 and 9) using a Guava easyCyte instrument (EMD Millipore) and replated at a 1:25 ratio to enable continuous culture.

#### Samples for RNA-seq analysis

The +Dox (Smad4 off) and −Dox (Smad4 on) cells were prepared as described immediately above. Then, 24 h after plating, cells were treated with either recombinant mouse TGFβ1 (100 pM; R&D Systems, 7666-MB-005) or TGFβ inhibitor SB505124 (2.5 µM; Sigma, S4696). Cells were harvested after 24 h of treatment.

### Tumor cell isolation

For in vivo RNA-seq, ATAC-seq and scMultiomics, tumor cells were freshly isolated from pancreata, livers or lungs of KC-shSmad4 mice by FACS. For in vitro RNA-seq and ATAC-seq, cells were harvested by trypsinization. For scRNA-seq of premalignant tissue, cells were freshly isolated from non-tumor-bearing KC-shRen and KC-shSmad4 pancreata by FACS after 48 h of cerulein or PBS treatment. Specifically, dissected tumors or premalignant pancreata were chopped and incubated in the following digestion buffer: 1 mg ml^−1^ collagenase V (Sigma, C9263), 2 U per ml Dispase II (Gibco, 17105041), 0.1 mg ml^−1^ DNase I (Sigma, DN25) and 0.1 mg ml^−1^ soybean trypsin inhibitor (STI; Sigma, T9003) in Mg^2+^/Ca^2+^-containing HBSS (Gibco, 14025076). Digestion was performed using the gentleMACS Octo dissociator (Miltenyi) for 42 min at 37 °C, followed by a PBS wash and an additional digestion with 0.05% trypsin–EDTA (Gibco, 15400) for 5 min at 37 °C. Samples were then treated with FACS buffer (2% FBS, 10 mM EGTA, 0.1 mg ml^−1^ DNase I and 0.1 mg ml^−1^ STI in PBS) to neutralize trypsin and then RBC lysis buffer (Invitrogen #00-4333-57) for 5 min at 20–25 °C, washed with FACS buffer (+DNase I/STI) and passed through a 100-μm strainer. Finally, these cells were resuspended in FACS buffer (+DNase I, STI and 300 nM DAPI) and filtered through a 40-μm strainer. Cells sorting was performed on MA900 (Sony), BD FACSAria I or BD FACSAria III (Becton Dickinson) instruments (gating scheme in Supplementary Fig. 1). For RNA-seq, cells were collected directly into TRIzol LS (Invitrogen, 10296028); for ATAC-seq, cells were collected in 2% FBS in PBS. For in vitro ATAC-seq, cell lines were harvested by trypsinization and sorted to yield identical number of cells for analysis as for the in vivo ATAC-seq.

### sWGS

Genomic DNA was freshly isolated and sWGS was performed essentially as previously described^64^. Libraries were sequenced on an Illumina HiSeq 4000 instrument. After mapping to the mouse genome and removal of duplicates, ~2.5 million reads were analyzed using the Varbin algorithm^65^.

### Bulk RNA-seq

#### RNA isolation, library preparation and sequencing

RNA was isolated using TRIzol LS (Invitrogen, 10296028) and column cleanup with an RNeasy kit (Qiagen, 74106). Following quantification (Nanodrop) and quality assessment (Agilent 2100 BioAnalyzer), 100–500 ng of RNA was selected using poly(A) and used for library preparation according to the manufacturer’s protocol (Illumina, 20020595), with 15 cycles of PCR. Samples were barcoded and subjected to a 75-bp single-end run on a HiSeq 4000 (Illumina) instrument, averaging ~50 million reads per sample.

#### RNA-seq read mapping, differential expression analysis and data visualization

Adaptor sequences were removed using Trimmomatic^66^. Reads were then aligned to GRCm38.91 (mm10) with STAR^67^ and transcripts were quantified with featureCounts^68^ to generate the raw count matrix. Differential gene expression and principal component analysis (PCA) were performed using the DESeq2 package^69^. DEGs were defined by a fold change > 2 with Benjamini–Hochberg-adjusted *P* value < 0.05. For heat map visualization, samples were normalized by *z* score and plotted with the ‘pheatmap’ package. For in vitro RNA-seq, DEGs were defined by intersecting upregulated genes after 24 h for −Dox versus +Dox in cells treated with TGFβ1 or SB505124 and removing overlapping genes to define SMAD4-dependent DEGs^11^.

#### Functional annotations of gene sets

Pathway enrichment analysis was performed using enrichR^70^. Significance was assessed using combined score, described as *c* = log(*P*) × *z*, where *P* is Fisher’s exact test *P* value and *z* is the *z* score for deviation from the expected rank, and adjusted *P* values. Combined scores of top enriched pathways from the ‘Kyoto Encyclopedia of Genes and Genomes (KEGG) 2019 Mouse’ database were compared between our in vitro (24 h) and in vivo (7 + 14 days combined) samples and Pearson correlation was used to assess significance.

#### Intersection of RNA-seq and ChIP-seq data

Two publicly available ChIP-seq datasets were used^11,24^. First, SMAD4-dependent SMAD2/3 ChIP-seq peaks that were significantly enriched in both studies (*P* < 10^−8^ and >8-fold enrichment of ChIP signal) were extracted. These peaks were then associated with genes on the basis of UCSC.mm10.knownGene using ChIPseeker^71^; they were analyzed for genic location (annotatePeaks) and the nearest transcription start site (TSS) was used to annotate the peak to that gene. The resulting gene list was intersected with SMAD4-dependent DEGs in the present study. The log_2_ fold change between Smad4 on and off in each organ was plotted and the type of genomic binding region was color-annotated.

### Bulk ATAC-seq

#### Cell preparation, transposition, library construction and sequencing

A total of 60,000 mKate2^+^ cells were FACS-isolated, washed with 50 μl of cold PBS, resuspended in 50 μl of cold lysis buffer^72^ and centrifuged for 10 min at 500*g* at 4 °C. The nuclear pellet was treated with Nextera Tn5 transposase for 30 min at 37 °C with shaking at 1,000 rpm according to the manufacturer’s protocol (Illumina, FC-121–1030). Transposed DNA was isolated using the DNA clean and concentrator kit (Zymo Research, D4013). Libraries were prepared with the NEBNext high-fidelity 2× PCR master mix (New England Biolabs, M0541). Library quality was assessed with the Bioanalyzer high-sensitivity DNA analysis kit (Agilent). Samples were subjected to a 150-bp paired-end run on a HiSeq 4000 (Illumina) instrument, averaging ~50 million reads per sample.

#### Mapping, peak calling and dynamic peak calling

FASTQ files were trimmed with trimGalore and cutadapt^73^, the filtered reads were aligned to mm10 with Bowtie2 (ref. ^74^) and peaks were called using MACS2 (ref. ^75^) and only kept for analysis if their *P* value was ≤0.001 and they were not on the ENCODE blacklist. To build a peak atlas, all peaks within 500 bp were combined, the mapped reads for each sample were quantified using featureCounts^68^ and normalization was performed using DESeq2 (ref. ^69^). Samples were normalized to 10 × 10^6^ mapped reads for comparison to DepthNorm. The normalization factors from DESeq2 (ref. ^76^) and BEDTools genomeCoverageBed^77^ were used to create normalized bigWig files. Dynamic ATAC peaks were called if they had an absolute log_2_ fold change ≥ 0.58 and false discovery rate (FDR) ≤ 0.1.

#### ATAC-seq heat map clustering

The dynamic peaks determined by comparing pancreas, liver and lung ± Dox (Smad4 off and on) were clustered using *z* scores and *k*-means clustering (*k* = 4 or 5) and plotted with ComplexHeatmap^78^.

#### TF motif enrichment analyses

Motif enrichment analysis was performed on differentially expressed ATAC peaks between liver and lung with the HOMER de novo motif discovery tool^79^ using the findMotifsGenome command with the parameters ‘size = given’ and ‘length = 8–12’. Motif enrichment scores were calculated for ATAC gain or loss regions between liver and lung ± Dox (Smad4 off and on) by applying the findMotifsGenome command with ‘size = given’ and ‘length = 8–12’ in each peak set.

#### Integration of ATAC-seq and RNA-seq data

For each organ, differential gene expression analysis between −Dox and +Dox was first performed individually (pancreas, liver or lungs). Next, the resulting gene lists were intersected to identify organ-specific Smad4-responsive targets. EnrichR^70^ was used to calculate enrichment scores for annotated TF targets using the ChEA_2016 database. RNA score was defined as the −log_10_(adjusted *P* value). Separately, HOMER was used to compare ATAC-seq data between Liver (+Dox) and Lung (+Dox) samples to identify differentially expressed peaks (DEPs). Custom TF motifs were curated by combining all the pairwise +Dox comparisons between any two organs and these motifs were then used to reannotate the DEPs to get consistent enrichment scores across known TFs. ATAC-score was defined as the −log_10_(*P* value). Finally, combined scores were calculated by multiplying the respective RNA and ATAC scores. To determine the net change in ATAC and RNA combined scores for KLF and RUNX in the pancreas, each TF’s combined score was calculated for each Dox condition, followed by subtraction of the +Dox from the −Dox score.

### Single-cell sequencing

#### ATAC-seq cell preparation, transposition, library construction and sequencing

The 10x Genomics Chromium Next GEM single-cell multiome reagent kit A (1000282) and ATAC kit A (1000280) were used according to the manufacturer’s protocol ‘nuclei isolation for single-cell multiome ATAC + gene expression sequencing’. In brief, cells (95% viability) were lysed for 4 min and resuspended in nuclei buffer (PN-2000207). Lysis efficiency and nucleus concentration were evaluated by trypan blue and DAPI staining (Countess II, Life Technologies). A total of 11,000 nuclei per transposition reaction were loaded, targeting the recovery of 7,000 nuclei after sequencing. Nuclei were encapsulated and barcoded; libraries were constructed following the manufacturer’s instructions and sequenced on an Illumina NovaSeq 6000 instrument.

#### Quality control and cell filtering

For scMultiomics (ATAC-seq + RNA-seq), nucleosome signal and TSS enrichment scores were computed. Retention criteria for individual cells were as follows: TSS enrichment score > 0.3, nucleosome signal score < 1.5, total ATAC-seq counts = 1,000–200,000 (based on the 10x Cell Ranger ATAC-seq count matrix) and total RNA counts = 1,000–50,000.

#### RNA-seq cell preparation, library construction and sequencing

The 10x Genomics Chromium instrument was used according to the manufacturer’s scRNA-seq protocol for 3′ v2. In brief, cells (>80% viability by trypan blue) were captured in droplets and subjected to reverse transcription and cell barcoding. Emulsions were broken and complementary DNA was purified with Dynabeads MyOne SILANE (Life Technologies) and PCR-amplified per the manufacturer’s instructions. The target cell number was ~5,000 per sample. Libraries were sequenced on Illumina NovaSeq 6000 S4 (R1, 28 cycles; i7, 8 cycles; R2, 90 cycles).

#### scRNA-seq data preprocessing and cell annotation

For both scMultiomics and scRNA-seq, gene expression UMI count data were normalized using SCTransform, percentage mitochondrial RNA content was regressed out and PCA was performed on the SCTransform Pearson residual matrix using the RunPCA function in Seurat^80,81^. Nearest neighbors were identified using FindNeighbors with dims = 1:30. The R package BBKNN was used to remove batch effects between samples and cell types were annotated using R packages celldex, SingleR and Azimuth with custom gene sets^80,81^. Only cells annotated as ductal or acinar were retained for downstream analysis.

#### scATAC-seq data preprocessing

scATAC-seq peaks were identified using MACS2 (ref. ^75^) with default parameters. Peak calling was performed using the CallPeaks function in Signac. Peaks overlapping annotated genomic blacklist regions for the mm10 genome were removed. Counts for the resulting peak set were quantified for each cell using the FeatureMatrix function in Signac. For scATAC-seq analysis, data were normalized using the RunTFIDF function and the top features were identified using FindTopFeatures with min.cutoff = ‘10’. The RunSVD function was used to create the linear selection index (LSI) space and the resulting visualization was generated with uniform manifold approximation and projection (UMAP) using dims = 2:30. Clusters were identified using the ‘algorithm = 3’ option.

#### Mapping of bulk ATAC-seq signatures

DEPs from bulk ATAC-seq were used to overlap with accessible peaks from scATAC-seq using intersectbed from BEDTools^77^. Peaks with at least 1-bp overlaps were kept and the top 5,000 scATAC-seq peaks sorted on the basis of significance were used to calculate ATAC signature scores using AddChromatinModule from Signac. LiverOPEN and LungOPEN cells were identified on the basis of the signature score and mutual exclusivity was calculated using the chi-squared test.

#### Differential gene expression of scRNA-seq data

FindMarkers function from Seurat^80,81^ with ‘min.pct = 0.1’ was used to identify DEGs in scRNA-seq data between LiverOPEN and LungOPEN cells defined on the basis of their matching scATAC-seq data. These DEGs were used for calculating gene signature scores for human PDAC scRNA-seq data using the AddModuleScore function from Seurat. Pathway enrichment analysis was also performed using these DEGs in enrichR^70^ with annotated pathways in the ‘KEGG 2019 Mouse’ database. Significance was assessed using combined scores and a lollipop plot was used to represent liver-enriched and lung-enriched pathways. To annotate premalignant cell types (gastric-like, progenitor-like, ADM, tuft and neuroendocrine), gene signatures were derived as follows. First, diffxpy (version 0.7.4, https://github.com/theislab/diffxpy) was used to conduct pairwise differential gene expression between established premalignant subpopulations^36^, using the Wald test with total counts per cell as a numeric covariate. Upregulated genes in each pairwise comparison were defined as those with *q* < 0.05 and log_2_ fold change > 1, excluding genes with mean expression < 0.05. A gene signature for a given subpopulation was defined as the set of genes that was upregulated in such subpopulation in every pairwise comparison against all other subpopulations.

#### Milo analysis of scRNA-seq data

A *k*-nearest-neighbor graph (*k* = 30) was constructed on the basis of the first 30 principal components using the buildGraph function in Milo^82^. Neighborhoods were identified with the makeNhoods function (prop = 0.1, refined = TRUE). Default parameters were applied for countCells, testNhoods and calcNhoodDistance to calculate statistical significance and apply spatial FDR correction. Visualization was performed using plotNhoodGraphDA (α = 0.1).

### Public scATAC-seq analysis

scATAC-seq data^35^ were retrieved from the Gene Expression Omnibus (GEO; GSE137069). Cells were kept using filters ‘min.cells > 10, min.features < 200’. Peaks that overlap with annotated genomic blacklist regions for the mm10 genome were identified. Cells were then retained using filters ‘peak_region_fragments > 3,000, peak_region_fragments < 20,000, pct_reads_in_peaks > 15, blacklist_ratio < 0.05, nucleosome_signal < 4, TSS.enrichment > 2’. Data were normalized using the RunTFIDF function and the top features were identified using FindTopFeatures. We used the RunSVD function to create the LSI space and the resulting visualization was generated with UMAP using dims = 2:30. Clusters were identified using the following options: ‘algorithm = 3’ and ‘resolution = 0.5’. GeneActivity from Signac was used to derive an approximate gene activity matrix and custom gene signatures were used to annotate cell types. Ductal and acinar cells were retained for downstream analysis. Differentially accessible peaks from bulk ATAC-seq were used to intersect with accessible peaks from scATAC-seq using intersectbed from BEDTools^77^. Peaks with at least 1-bp overlap were kept and the top 5,000 scATAC-seq peaks sorted on the basis of significance were used to calculate ATAC signature scores using AddChromatinModule from Signac.

### Human PDAC analysis

#### Metastasis recurrence analysis

Previously reported data^25,26^ were reanalyzed by site of recurrence (liver or lungs) and annotation of SMAD4 IHC status (positive or negative).

#### MSK-IMPACT analysis

Human datasets were obtained through the MSK Clinical Sequencing Cohort (MSK-IMPACT) using cBioPortal^83,84^. Samples were selected as follows: cancer type, pancreatic cancer; cancer type detailed, PDAC; genotype, SMAD4: MUT HOMDEL. Comparison of genomic annotations between SMAD4-altered and unaltered samples, along with corresponding statistical analyses, were generated and visualized using the cBioPortal.

#### Bulk transcriptomic analysis

Microarray data^40^ were retrieved from the GEO (GSE71729). First, we compared liver or lung metastasis samples to primary tumor samples to derive liver or lung metastasis-specific DEGs. Second, we performed analogous comparison of normal liver or lung versus normal pancreas samples. The tumor and normal gene sets were then intersected to identify liver and lung tumor-specific signatures by filtering out DEGs in the normal organs. Pathway enrichment analysis was performed on the resulting organ-specific or tumor-specific gene sets using enrichR^70^ and the top enriched TFs were plotted using combined scores in bar plot format.

#### scRNA-seq analysis

scRNA-seq data^37^ were retrieved from the GEO (GSE155698). Cells were retained using filters ‘min.cells > 100, nFeature_RNA > 500, nCount_RNA > 2,500, percent.mt < 25’. SCTransform was used to regress out the percentage of mitochondrial RNA and nearest neighbors were found using FindNeighbors with dims = 1:30. Clusters were identified using resolution = 0.8 and cell types were annotated using R packages celldex, SingleR and Azimuth with custom gene sets^80,81^. Only ductal and acinar cells were retained for calculating gene signature scores from our mouse multiomic data using AddModuleScore from Seurat^80,81^. FeaturePlot was used to visualize the differential peak openings from our mouse LiverOPEN and LungOPEN ATAC signatures, as well as gene signatures for gastric-like and progenitor-like cells from previously published datasets^36^. The Pearson correlation coefficient was used to quantify the relationship between these signature scores.

#### IHC analysis

Biopsied or surgically resected primary tumors, liver and lung metastases from 17 PDAC patients were obtained under a biospecimen research protocol approved by the MSKCC Institutional Review Board (protocol 22-159). All participants provided preprocedure informed consent. Participants were not compensated. Participants were both male and female, with an age range of 55–82 years at recruitment. Archival formalin-fixed, paraffin-embedded clinical tissue blocks were identified by database search and chart review and procurement was overseen by an expert pathologist (U.B.). IHC staining was performed as described above and signal intensity was estimated on a scale of 0 to 5 (none to strongest signal).

### Statistics and reproducibility

Statistical analyses were performed with GraphPad Prism (version 9), R (version 4.3.1) and Python (version 3.6.4). Sample size, error bars, statistical methods and exact *P* values are reported in each figure and/or associated legend. No statistical methods were used to predetermine sample sizes but our sample sizes are similar to those reported in previous publications^21,35,36^. No data were excluded from the analysis, unless specifically noted. For Smad4 restoration experiments, mice were randomized into +Dox versus −Dox groups. For IHC and IF experiments, random fields of view were used for quantification as described above. The other experiments were not randomized. The investigators were not blinded to allocation during experiments and outcome assessment, unless specifically noted. For pairwise comparisons, the Kolmogorov–Smirnov test was used to compare distributions and determine the appropriate test for comparison of means or medians (*t*-test or Mann–Whitney *U*-test). For other analyses, data distribution was assumed to be normal but this was not formally tested.

### Reporting summary

Further information on research design is available in the Nature Portfolio Reporting Summary linked to this article.

## Supplementary information

Supplementary Information Representative gating strategy for FACS-based isolation of SMAD4 off (a) and SMAD4 on (b) tumor cells.

Reporting Summary

Supplementary Table 1 Complete annotated lists of DEGs at the indicated organs and time points after Smad4 restoration.

Supplementary Table 2 Complete GO analysis for the indicated conditions after Smad4 restoration.

Supplementary Table 3 Complete list of differentially accessible peaks from bulk ATAC-seq data.

Supplementary Table 4 Complete GO analysis of liver and lung metastasis-like cell populations from scMultiome data.

Supplementary Table 5 Primer sequences.

## Source data

Source Data Fig. 1 and Extended Data Figs. 4 and 10 Uncropped western blot scans.

Source Data Figs. 1–8 and Extended Data Figs. 1–10 Numerical source data.

## Data Availability

All newly generated sequencing datasets were deposited and made publicly available from the GEO under accession code GSE245827. Previously published datasets are publicly available under the following accession numbers: sWGS, PRJNA866212; ChIP-seq, GSE72069 and GSE118765; scATAC-seq, GSE132330; human scRNA-seq, GSE155698; human microarray, GSE71729. Human PDAC genomic data were accessed and analyzed using cBioPortal for cancer genomics (https://www.cbioportal.org). All other data supporting the findings of this study are available from the corresponding authors on reasonable request. Source data are provided with this paper.

## Code availability

No unique code was developed for this study.
