# Supplementary material for: SMAD4 induces opposite effects on metastatic growth from pancreatic tumors depending on the organ of residence
Source: Nat Cancer. 2025 Sep 25;6(11):1839–56. doi: 10.1038/s43018-025-01047-5 (PMC12643927; doi:10.1038/s43018-025-01047-5)

nature cancer

Supplementary information

https://doi.org/10.1038/s43018-025-01047-5

SMAD4 induces opposite effects on metastatic growth from pancreatic tumors depending on the organ of residence

In the format provided by the authors and unedited

Supplementary Figure 1

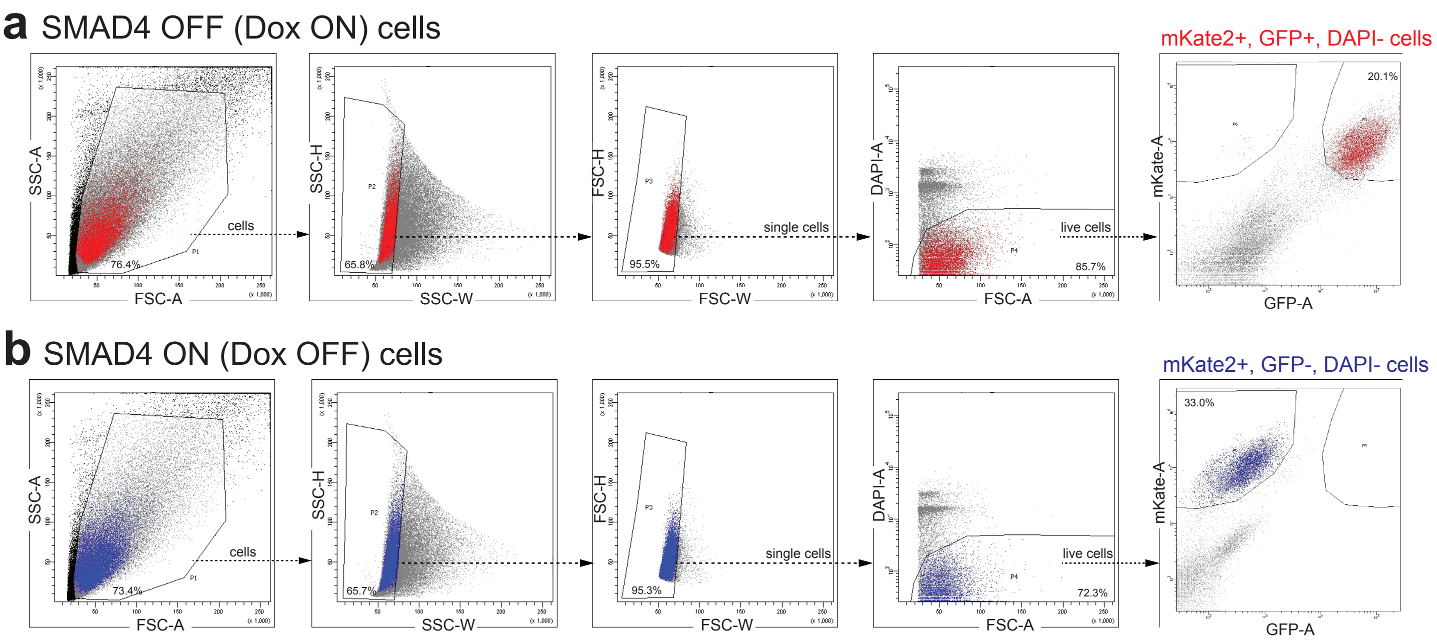

Supplement: Supplementary file 1 — Representative gating strategy for FACS-based isolation of SMAD4 off (a) and SMAD4 on (b) tumor cells. [file 43018_2025_1047_MOESM1_ESM.pdf]
