# Supplementary material for: SMAD4 induces opposite effects on metastatic growth from pancreatic tumors depending on the organ of residence
Source: Nat Cancer. 2025 Sep 25;6(11):1839–56. doi: 10.1038/s43018-025-01047-5 (PMC12643927; doi:10.1038/s43018-025-01047-5)
Supplement: Supplementary file 2 — Reporting Summary [file 43018_2025_1047_MOESM2_ESM.pdf]

nature portfolio

Corresponding author(s): Scott W. Lowe & Kaloyan M. Tsanov

Last updated by author(s): Jul 30, 2025

# Reporting Summary

Nature Portfolio wishes to improve the reproducibility of the work that we publish. This form provides structure for consistency and transparency in reporting. For further information on Nature Portfolio policies, see our Editorial Policies and the Editorial Policy Checklist.

## Statistics

For all statistical analyses, confirm that the following items are present in the figure legend, table legend, main text, or Methods section.

| n/a | Confirmed | |
|---|---|---|
| ☐ | ☒ | The exact sample size ($n$) for each experimental group/condition, given as a discrete number and unit of measurement |
| ☐ | ☒ | A statement on whether measurements were taken from distinct samples or whether the same sample was measured repeatedly |
| ☐ | ☒ | The statistical test(s) used AND whether they are one- or two-sided<br>*Only common tests should be described solely by name; describe more complex techniques in the Methods section.* |
| ☒ | ☐ | A description of all covariates tested |
| ☐ | ☒ | A description of any assumptions or corrections, such as tests of normality and adjustment for multiple comparisons |
| ☐ | ☒ | A full description of the statistical parameters including central tendency (e.g. means) or other basic estimates (e.g. regression coefficient) AND variation (e.g. standard deviation) or associated estimates of uncertainty (e.g. confidence intervals) |
| ☐ | ☒ | For null hypothesis testing, the test statistic (e.g. $F$, $t$, $r$) with confidence intervals, effect sizes, degrees of freedom and $P$ value noted<br>*Give P values as exact values whenever suitable.* |
| ☒ | ☐ | For Bayesian analysis, information on the choice of priors and Markov chain Monte Carlo settings |
| ☒ | ☐ | For hierarchical and complex designs, identification of the appropriate level for tests and full reporting of outcomes |
| ☐ | ☒ | Estimates of effect sizes (e.g. Cohen's $d$, Pearson's $r$), indicating how they were calculated |

*Our web collection on statistics for biologists contains articles on many of the points above.*

## Software and code

Policy information about availability of computer code

| | |
|---|---|
| Data collection | Experimental measurement, imaging and next-generation sequencing data were collected using standard methods, as described in detail in the Methods section of the manuscript. Animal imaging data were collected using Vevo 2100 System (VisualSonics) for ultrasound and IVIS Spectrum imager (Perkin Elmer) for bioluminescence; tissue imaging data were collected using SMZ1500 stereomicroscope with NIS-Element v3.0 (Nikon) and AxioImager microscope with ZEN 3.3 (Zeiss); flow cytometry data were collected on a BD FACSAria I or BD FACSAria III cell sorter with FACS Diva (Becton Dickinson), or MA900 (Sony) cell sorter; next-generation sequencing data were collected using HiSeq 4000 and NovaSeq 6000 systems (Illumina). |
| Data analysis | Data were analyzed using commercially available and open-source software, as specified in the Methods section of the manuscript. Animal imaging data were analyzed using Vevo LAB v3.2 (VisualSonics) for ultrasound and Living Image v4.5 (Perkin Elmer) for bioluminescence; tissue imaging data were analyzed using Fiji/ImageJ (v.2.0); next-generation sequencing data were analyzed using R (v4.3.1) and Python (v.3.6.4); statistical analyses were performed using GraphPad Prism (v.9). No unique code was developed for this study, and the employed algorithms have been references in the Methods.. |

For manuscripts utilizing custom algorithms or software that are central to the research but not yet described in published literature, software must be made available to editors and reviewers. We strongly encourage code deposition in a community repository (e.g. GitHub). See the Nature Portfolio guidelines for submitting code & software for further information.

## Data

Policy information about availability of data

All manuscripts must include a data availability statement. This statement should provide the following information, where applicable:

- Accession codes, unique identifiers, or web links for publicly available datasets
- A description of any restrictions on data availability
- For clinical datasets or third party data, please ensure that the statement adheres to our policy

All newly generated sequencing datasets have been deposited and made publicly available under GEO under accession code GSE245827. Previously published datasets are publicly available under the following accession numbers: sWGS (PRJNA866212); ChIP-seq (GSE72069, GSE118765); scATAC-seq (GSE132330); human scRNA-seq (GSE155698); human microarray (GSE71729). Human PDAC genomic data were accessed and analyzed using the cBioPortal for cancer genomics (https://www.cbioportal.org). All numerical source data have been provided in a Source Data Table, and uncropped Western blots in a Source Data file.

## Research involving human participants, their data, or biological material

Policy information about studies with human participants or human data. See also policy information about sex, gender (identity/presentation), and sexual orientation and race, ethnicity and racism.

| | |
|---|---|
| Reporting on sex and gender | Patients were both male and female. Sex and gender were not considered in study design due to the small overall number of patients, so we refrain from drawing conclusions regarding the influence of sex and gender on the presented data. |
| Reporting on race, ethnicity, or other socially relevant groupings | Race, ethnicity and other socially relevant groupings were not considered in study design due to the small overall number of patients, so we refrain from drawing conclusions regarding the influence of these factors on the presented data. |
| Population characteristics | No data on population characteristics was collected or used. |
| Recruitment | Patients receiving standard care at MSKCC donated their tumor samples according to an approved biospecimen research protocol. Samples analyzed in this study were selected based on the availability of a sufficient amount of archival FFPE tumor tissue from patients who had given their consent to tissue utilization for research purposes. |
| Ethics oversight | Archival human specimens were procured under the approved MSKCC Institutional Review Board biospecimen research protocol #22-159. All patients provided pre-procedure written informed consent. |

Note that full information on the approval of the study protocol must also be provided in the manuscript.

# Field-specific reporting

Please select the one below that is the best fit for your research. If you are not sure, read the appropriate sections before making your selection.

☒ Life sciences ☐ Behavioural & social sciences ☐ Ecological, evolutionary & environmental sciences

For a reference copy of the document with all sections, see nature.com/documents/nr-reporting-summary-flat.pdf

# Life sciences study design

All studies must disclose on these points even when the disclosure is negative.

| | |
|---|---|
| Sample size | No statistical methods were used to predetermine sample sizes but our sample sizes are similar to those reported in previous publications (PMID: 33536616, 37167403, 36344707, 38177458). |
| Data exclusions | No data were excluded from the analysis, unless specifically noted in the text. |
| Replication | Experiments were replicated using independent cell lines and mice, as specified in the respective figure legends. |
| Randomization | For Smad4 restoration experiments, mice were randomized into +Dox vs. –Dox groups. For IHC/IF experiments, random fields of view were used for quantification as described in the Methods. The rest of the experiments were not randomized; to control for covariates, the compared groups were identical/isogenic except for the tested variable (gene knockdown, treatment, or organ of residence). |
| Blinding | For mouse experiments, the investigators were not blinded to group allocation and outcome assessment, since mice had to be fed different chow (+Dox or -Dox). For IHC/IF analysis, the investigators were blinded during data acquisition and analysis as indicated in the Methods. For the rest of the experiments, blinding was not possible because of the need for different treatment or comparison between different organs. |

# Reporting for specific materials, systems and methods

We require information from authors about some types of materials, experimental systems and methods used in many studies. Here, indicate whether each material, system or method listed is relevant to your study. If you are not sure if a list item applies to your research, read the appropriate section before selecting a response.

## Materials & experimental systems

| n/a | Involved in the study | |
|---|---|---|
| ☐ | ☒ | Antibodies |
| ☐ | ☒ | Eukaryotic cell lines |
| ☒ | ☐ | Palaeontology and archaeology |
| ☐ | ☒ | Animals and other organisms |
| ☒ | ☐ | Clinical data |
| ☒ | ☐ | Dual use research of concern |
| ☒ | ☐ | Plants |

## Methods

| n/a | Involved in the study | |
|---|---|---|
| ☒ | ☐ | ChIP-seq |
| ☐ | ☒ | Flow cytometry |
| ☒ | ☐ | MRI-based neuroimaging |

# Antibodies

**Antibodies used**

For IHC/IF, the following primary antibodies were used: SMAD4 (clone EP618Y, Millipore #04-1033, 1:200, IHC), pSMAD2 (clone 138D4, Cell Signaling #3108, 1:100, IHC), p57 (Atlas Antibodies #HPA002924, 1:500, IHC), KLF4 (Abcepta #AM2725A, 1:100, IHC), KLF5 (Abcam #ab137676, 1:500, IHC), KLF6 (Abcam #ab241385, 1:1,000, IHC), RUNX1 (clone D4A6, Cell Signaling #8529, 1:500, IHC), RUNX2 (clone D1L7F, Cell Signaling #12556, 1:500, IHC), RUNX3 (clone 2B3, Life Technologies #MA5-17169, 1:500, IHC), mKate2 (Evrogen #AB233, 1:1,000, IF), Ki67 (clone B56, BD Pharmingen #550609, 1:200, IF), □□SMA (clone 1A4, Sigma #A2547, 1:1,000, IF).

For IHC, HRP-conjugated secondary antibodies (ImmPRESS kits, Vector Laboratories #MP7401 and #MP2400) were used and visualized with DAB substrate (ImmPACT kit, Vector Laboratories #SK-4105). For IF, secondary Alexa Fluor 488 (#A-21202) or 594 (#A-21207) dye-conjugated antibodies (Life Technologies, 1:500) were used.

For Western blot, the following primary antibodies were used: SMAD4 (clone B-8, Santa Cruz #sc-7966, 1:500), p57 (Atlas # HPA002924, 1:1,000), KLF4 (Abcepta #AM2725A, 1:1000), RUNX1 (Cell Signaling #8529, 1:1000), and Actin-HRP (clone AC-15, Sigma #A3854, 1:20,000).

**Validation**

All antibodies have been validated by the manufacturer (links below and references therein) and/or in prior publications and/or by shRNA knockdowns in the current study:

SMAD4, WB: https://www.scbt.com/p/smad4-antibody-b-8. Additionally validated by shRNA knockdown in the present study.
SMAD4, IHC: https://www.fishersci.com/shop/products/anti-smad4-clone-ep618y-millipore/50171160.
pSMAD2, IHC: https://www.cellsignal.com/products/primary-antibodies/phospho-smad2-ser465-467-138d4-rabbit-mab/3108.mKate2, IF: https://evrogen.com/products/antibodies/AB-tRFP.shtml (references PMID: 33536616, 37167403).
p57, IHC: https://www.atlasantibodies.com/products/primary-antibodies/triple-a-polyclonals/anti-cdkn1c-antibody-hpa002924. Additionally validated by shRNA knockdown in the present study.
KLF4, IHC: https://www.abcepta.com/products/AP2725a-KLF4-Antibody-N-term-C74. Additionally validated by shRNA knockdown in the present study.
KLF5, IHC: https://www.abcam.com/en-us/products/primary-antibodies/klf5-antibody-ab137676.
KLF6, IHC: https://www.abcam.com/en-us/products/primary-antibodies/klf6-antibody-ab241385.
RUNX1, IHC & WB: https://www.cellsignal.com/products/primary-antibodies/aml1-d4a6-rabbit-mab/8529.Additionally validated by shRNA knockdown in the present study.
RUNX2, IHC: https://www.cellsignal.com/products/primary-antibodies/runx2-d1l7f-rabbit-mab/12556.
RUNX3, IHC: https://www.thermofisher.com/antibody/product/RUNX3-Antibody-clone-2B3-Monoclonal/MA5-17169.
Actin-HRP, WB: https://www.sigmaaldrich.com/US/en/product/sigma/a3854.
Ki67, IF: https://www.bdbiosciences.com/en-us/products/reagents/flow-cytometry-reagents/research-reagents/single-color-antibodies-ruo/purified-mouse-anti-ki-67.550609.
a-SMA, IF:https://www.sigmaaldrich.com/US/en/product/sigma/a2547.

# Eukaryotic cell lines

Policy information about cell lines and Sex and Gender in Research

| | |
|---|---|
| Cell line source(s) | Primary cell lines were derived from male mouse tumor tissues, as described in the Methods. |
| Authentication | Primary cultures were authenticated by flow cytometry of engineered fluorescent alleles. |
| Mycoplasma contamination | All cultures were tested negative for mycoplasma. |
| Commonly misidentified lines (See ICLAC register) | No commonly misidentified lines were used in this study. |

## Animals and other research organisms

Policy information about studies involving animals; ARRIVE guidelines recommended for reporting animal research, and Sex and Gender in Research

| | |
|---|---|
| Laboratory animals | Mice (Mus musculus) were maintained under pathogen-free conditions, housed on a 12h–12h light–dark cycle under ambient temperature of 18–24°C and 40–60% humidity. Food and water were provided ad libitum.<br><br>Ptf1aCre/+;LSL-KrasG12D/+;Rosa26LSL-rtTA3-IRES-mKate2/+(RIK);Col1a1shRNA-Homing-Cassette/+(CHC) male embryonic stem cells (ESCs) (C57BL/6J; 129 mixed background) were targeted with two independent GFP-linked Smad4 shRNAs (shSmad4.591: CAAAGATGAATTGGATTCTTT; shSmad4.1599: ACAGTTGGAATGTAAAGGTGA). The KC-shRen ESC control clone used in this study has been described [PMID: 24395249]. Whenever applicable, Dox treatment was started at 5-6 weeks of age. Cerulein treatment was done at 8 weeks of age.<br><br>Transplantation experiments (orthotopic and experimental metastasis assays) were performed in 6-8 week-old female Foxn1nu (athymic nude) mice. |
| Wild animals | No wild animals were used in this study. |
| Reporting on sex | GEMMs were male since the ESCs used to generate them are male. For transplantation experiments, female hosts were used as recipients, as it greatly reduces the cost and complications of housing adult male animals in the same cage. |
| Field-collected samples | No field-collected samples were used in this study. |
| Ethics oversight | All mouse experiments were approved by the Memorial Sloan Kettering Cancer Center Institutional Animal Care and Use Committee (IACUC) under Protocol #11-06-018. |

Note that full information on the approval of the study protocol must also be provided in the manuscript.

## Plants

| | |
|---|---|
| Seed stocks | Not relevant to this study. |
| Novel plant genotypes | Not relevant to this study. |
| Authentication | Not relevant to this study. |

## Flow Cytometry

### Plots

Confirm that:

☒ The axis labels state the marker and fluorochrome used (e.g. CD4-FITC).

☒ The axis scales are clearly visible. Include numbers along axes only for bottom left plot of group (a 'group' is an analysis of identical markers).

☒ All plots are contour plots with outliers or pseudocolor plots.

☒ A numerical value for number of cells or percentage (with statistics) is provided.

### Methodology

| | |
|---|---|
| Sample preparation | Described in detail under 'Tumor cell isolation' in the Methods. |
| Instrument | Cells were sorted on a BD FACSAria I, BD FACSAria III (Becton Dickinson), or MA900 (Sony) cell sorter. |
| Software | Flow cytometry data was collected and analyzed using the BD FACS Diva or Sony MA900 software. |
| Cell population abundance | Representative data are provided in Supp. Fig. 1. Population purity was assessed by using the fluorescent reporters engineered in the models. |

| Gating strategy | Provided in Supp. Fig. 1. |
|---|---|

☒ Tick this box to confirm that a figure exemplifying the gating strategy is provided in the Supplementary Information.
