# Supplementary figures and images for: SMAD4 induces opposite effects on metastatic growth from pancreatic tumors depending on the organ of residence
Source: Nat Cancer. 2025 Sep 25;6(11):1839–56. doi: 10.1038/s43018-025-01047-5 (PMC12643927; doi:10.1038/s43018-025-01047-5)

# Source Data: Uncropped scans of Western blots

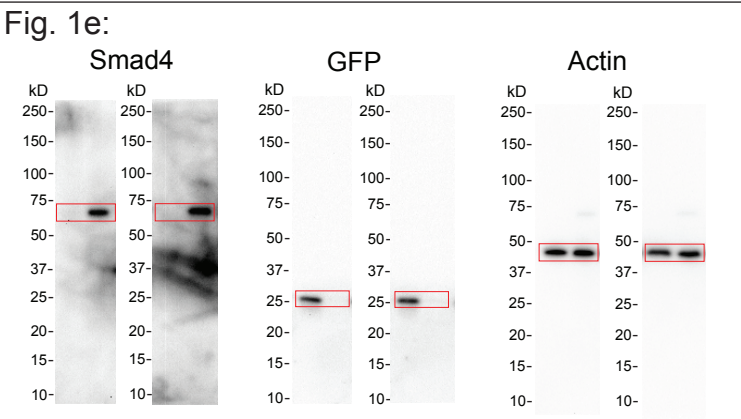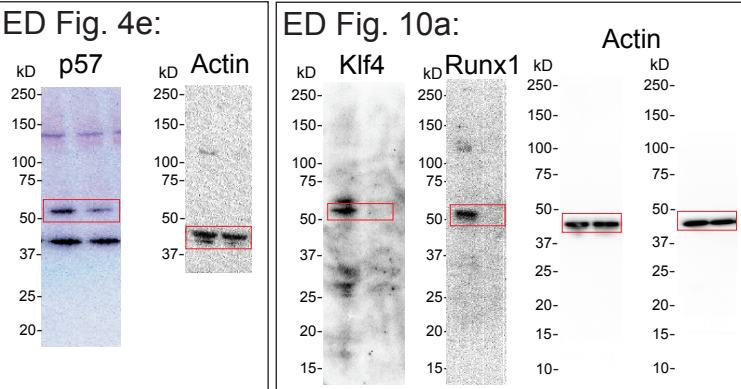

Supplement: Supplementary file 8 — Uncropped western blot scans. [file 43018_2025_1047_MOESM8_ESM.pdf]
